# Design and evaluation of a smart passive dynamic arm support for robotic-assisted laparoscopic surgery

**DOI:** 10.1007/s11701-024-01820-1

**Published:** 2024-02-10

**Authors:** Pim Schrijvershof, A. Masie Rahimi, Nicola Leone, EAES TC Robotics, Alexander Bloemendaal, Freek Daams, Alberto Arezzo, Yoav Mintz, Tim Horeman

**Affiliations:** 1 Department of Biomechanical Engineering, Technical University of Delft, Delft, The Netherlands; 2 Department of Surgery, Amsterdam UMC – VU University Medical Center, Amsterdam, The Netherlands; 3 Amsterdam Skills Centre for Health Sciences, Amsterdam, The Netherlands; 4 Cancer Center Amsterdam, Amsterdam, The Netherlands; 5 Department of Surgical Sciences, University of Torino, Torino, Italy; 6 Reinier de Graaf Hospital, Delft, The Netherlands; 7 Department of General Surgery, Hadassah Hebrew-University Medical Center, Jerusalem, Israel

**Keywords:** Robotic surgery, Ergonomics, Arm support, RAS, Laparoscopy, Fatigue

## Abstract

Surgeons performing robotic-assisted laparoscopic surgery experience physical stress and overuse of shoulder muscles due to sub-optimal arm support during surgery. The objective is to present a novel design and prototype of a dynamic arm support for robotic laparoscopic surgery to evaluate its ergonomics and performance on the AdLap-VR simulation training device. The prototype was designed using the mechanical engineering design process: Technical requirements, concept creation, concept selection, 3D-design and built of the prototype. A crossover study was performed on a marble sorting task on the AdLap-VR. The first group performed four trials without the arm support, followed by four trials with the arm support, and the other group executed the sequence vice versa. The performance parameters used were time to complete (s), path length (mm), and the number of collisions. Afterward, the participants filled out a questionnaire on the ergonomic experience regarding both situations. 20 students executed 160 performed trials on the AdLap-VR Significant decreases in the subjective comfort parameters mental demand, physical demand, effort and frustration were observed as a result of introducing the novel arm support. Significant decreases in the objective performance parameters path length and the number of collisions were also observed during the tests. The newly developed dynamic arm support was found to improve comfort and enhance performance through increased stability on the robotic surgery skills simulator AdLap-VR.

**Supplementary Information:**

The online version contains supplementary material available at 10.1007/s11701-024-01820-1.

## Introduction

Developments in robotic-assisted laparoscopic surgery (RALS) have been made to enable more precise surgical movements and gestures while minimizing invasiveness and recovery time for patients [1]. RALS, in addition to bringing practical surgical benefits, has improved surgeon comfort by relieving them of some of the physical stress associated with traditional laparoscopy, such as injuries and overuse of the neck, shoulders, and lower back muscles [2]. With RALS, surgeons can perform advanced laparoscopy with steerable instruments while maintain a more comfortable position.

Although RALS has improved surgeon comfort by reducing the strain on the surgeon’s body during laparoscopic surgery, ergonomic assessments and studies have revealed that there are still ergonomic risks associated with robotic laparoscopy [3–5]. One potential risk in the current surgical setup is related to the arm support of the master device, which typically includes a fixed leather arm pad positioned in front of the surgeon. In a 2016 study by Yang [6], researchers found that surgeons’ arms are frequently unsupported during surgery due to the fixed nature of the arm support. This is caused by the limited range of motion that can be achieved while resting elbows on the arm support, forcing surgeons to leave the arm support to adjust instrument positions using the clutch system of the da Vinci Surgical System. This leads to increased muscle activity in the shoulders and trapezius. Additionally, the fixed leather arm pad has limited ability to provide support beyond the elbow region, resulting in increased biceps fatigue as the surgeon’s forearms must be constantly supported. These limitations could compromise the comfort and performance of the surgeon during extended periods of surgery [7].

A literature review is presented in Supplemental file 1, covers all passive dynamic arm support systems found in the literature. It presents 108 different arm support systems, how they function, and classifies the various working principles. The report discusses the most promising working principles for RALS and identified the systems that best fit the type of applications within RALS such as suturing and threading. Although it was found that there are already devices that are able to support the arms of its user and even arm supports made for open surgery, there is no dynamic arm support yet specifically designed for robotic-assisted surgery. For the applications within robotic-assisted laparoscopic surgery, it is estimated that 4-bar mechanisms with an added lever are the most promising, with 4-bar mechanisms with the base as a vertical linkage to be the most useful for horizontal movement applications, and 4-bar mechanisms without the base as a vertical linkage are deemed to be the most useful for vertical movement applications. Therefore, the goal of this design and validation study is to create a dynamic arm support for surgeons with position tracking of the arm supports and to evaluate it with a study on users performing RALS simulation tasks with and without the designed arm support.

## Methods

The Bare Minimum Design approach (BMD) was used to facilitate a more structured development of the prototype [8]. As a first step in the design process, the problem was translated into a list of technical requirements and performance criteria. Subsequently, the design process for the arm support and sensors was discussed. Finally, we explained the study design used to validate the prototype.

### Technical requirements & performance criteria

(1) The device should be able to support user arms weighing between 2.8 and 5.4 kg, which corresponds to 5.3% of the weight of individuals ranging from 54 to 101 kg (average weight women −25% – average weight men + 25%) [9]. (2) The device should be strong enough to withstand a load of 11 kg on each arm support without plastic deformation, equivalent to twice the weight of a 101 kg person’s arm. (3) The device should allow the required range of motion (ROM) for using the AdLap-RS system [10], with ROM specifications as follows: For the elbow: 20 cm in the x-axis, 42 cm in y-axis, and 20 cm in the z-axis. For the wrist: 31 cm in the x-axis, 46 cm in the y-axis, and 20 cm in the z-axis. (4) The arm support should not negatively influence performance on the AdLap-VR for the parameters of time, path length, and the number of collisions. (5) The arm support should be adaptable for use in both standing and seated postures. (6) Users should be able to install and remove the arm support within 30 s. (7) When not in use, the arm support should be folded away without any hindrance, with the maximum protruding distance from the AdLap-VR case at all sides not exceeding 15 cm. (8) The positions of the points of contact of the arm support with the arms of the user should be measurable with an accuracy of 10 mm and a step sensitivity of 2 mm.

Potential design solutions were validated based on the following performance criteria. (1) Comfort, the higher the comfort level, the better the scores. (2) Cost of materials. (3) Complexity, measured by the number of moving parts, (4) Smoothness of motion during use. (5) Intuitiveness, easier setup and use result in a higher score. (6) Volume, a lower system volume during transport and use is given a higher score. (7) Durability, a maintenance friendly system that is less prone to errors receives a betters score.

### Design strategy

**Fig. 1:**
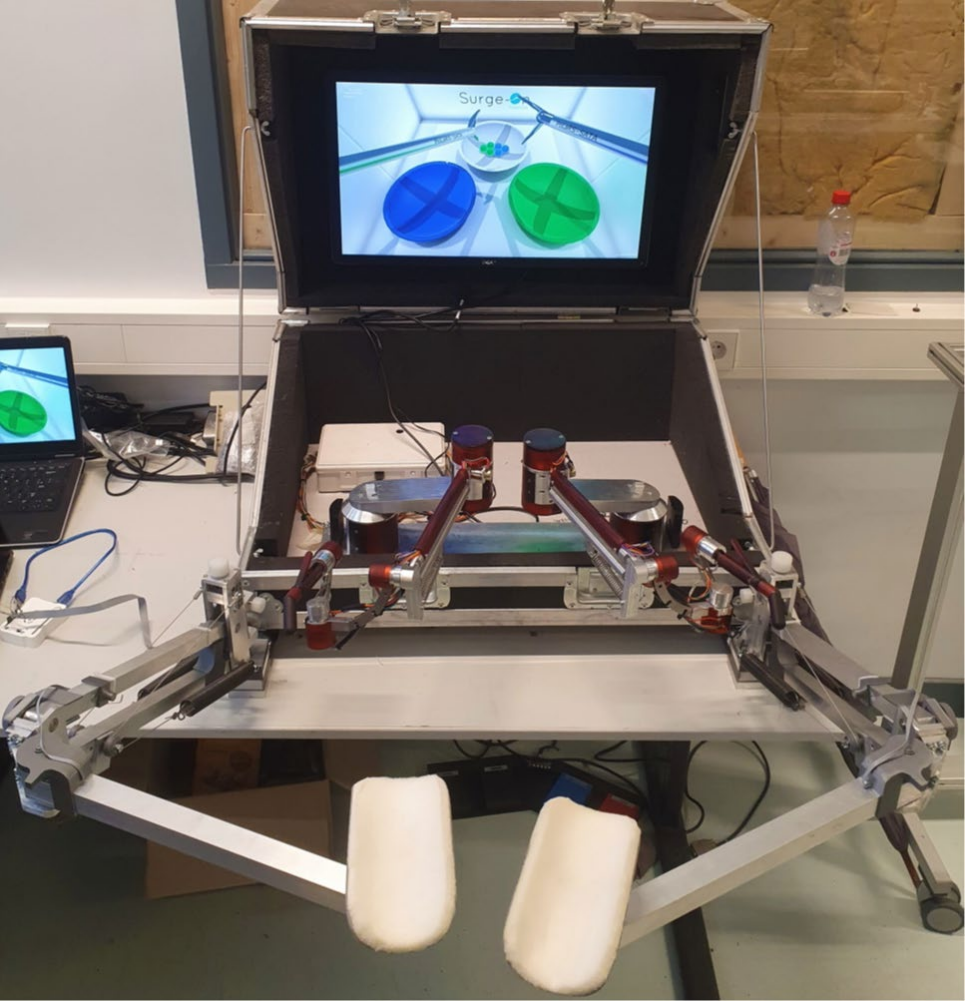
Setup of the AdLap-RS [10] with the newly developed Dynamic Arm Support

In line with the BMD process for surgical devices [8], a morphological chart was created that comprises sub-solutions for partial system functions. Within the chart, three design routes were identified that can lead to a potential system. One is the most durable, one leads to the most compact design, and one to the most adjustable system. These concepts were assessed using a Harris profile and the best concept was worked out in full detail (Supplemental file 2). All structural parts were evaluated through simulations using Final Element software (Solidworks v.2022, SW corporation, Waltham, Massachusetts, USA) before being manufactured into a functional prototype. To track the opposition of the arms, new rotations sensors were developed based on the magnetic hall effect. Supplemental file 2 shows how these sensors were developed, integrated and validated in order to track the position of the arm supports. Figure 1 shows the complete system as being used during the experiments.

### Study protocol

**Fig. 2:**
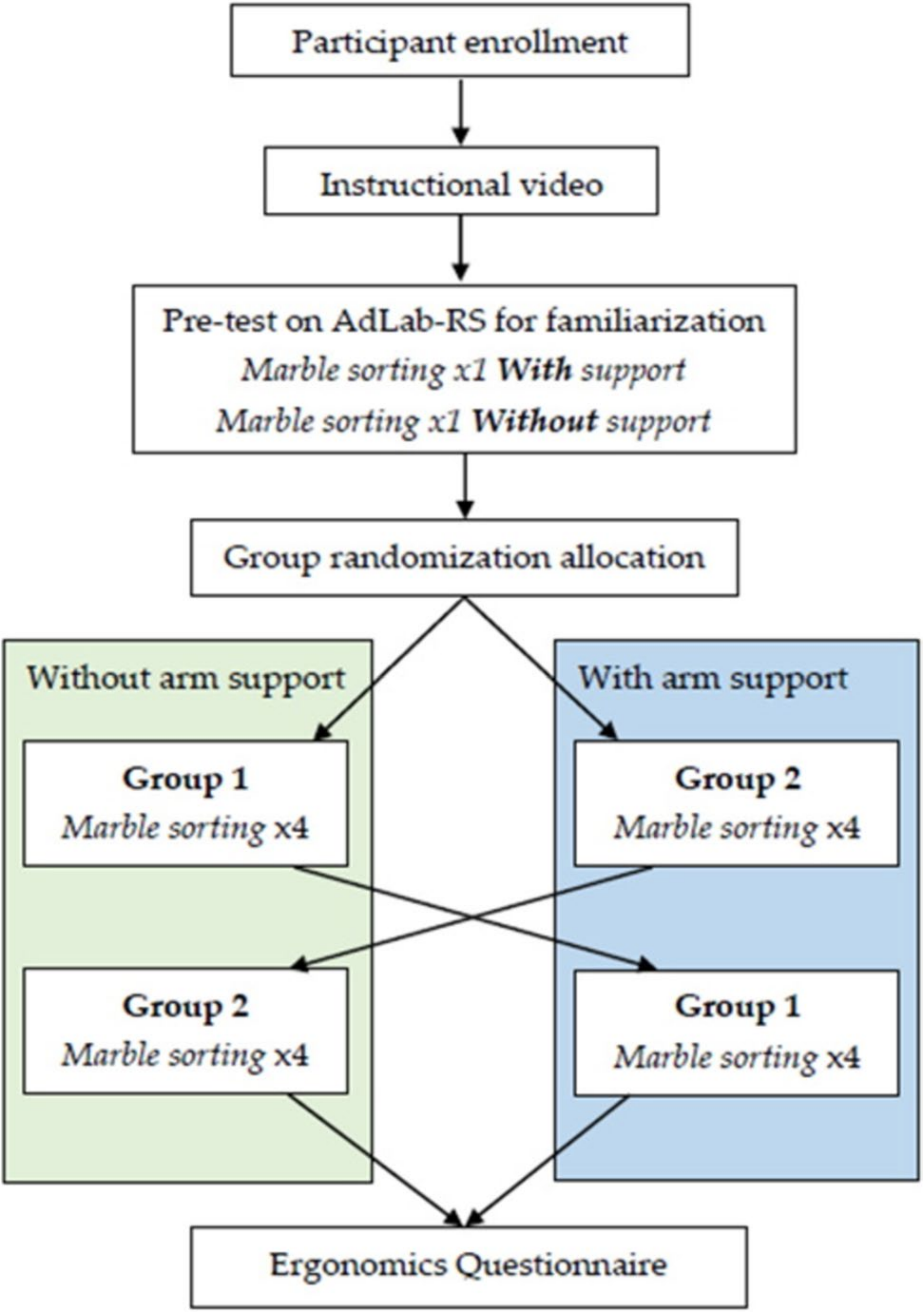
Flowchart of the crossover study design

Biomechanical Students at the Delft University of Technology were recruited for voluntary participation in the study as novices. All participants were first shown an instructional video of the task to ensure a baseline of equal information prior to the tests. A pre-test was conducted on the AdLap-VR to familiarize participants with the system’s inputs and the digital environment. The participants were then randomized into two groups for a crossover study (Fig. 2). Both groups completed a single exercise on the AdLap-VR system for a total of eight times. The first group conducted the first four trials without arm support, followed by four trials with arm support. The second group did the first four trials with arm support, followed by four trials without arm support.

### Training task

In the exercise, participants were asked to sort marbles by color into matching bowls. They were instructed to sort the green marbles with their left hand and the blue marbles with their right hand to ensure bimanual performance, and alternating left and right to ensure no differences in strategy. After completing the tests, participants filled out a questionnaire and provided feedback on their experience using the AdLap-VR with and without the arm support system. The questionnaire focused mainly on the system’s ergonomics. It included questions scored from 1 to 20 formatted according to the NASA-TLX questionnaire [11], combined with open questions regarding their experience (Supplemental file 3).

### Performance parameters

The main performance parameters from the AdLap-VR in this study included time (s) to complete the task, total instrument path length (mm), and the number of collisions. A time limit of 5 min was set for each trial for logistical reasons, and trials exceeding this time limit were marked as “did not finish” and excluded from the analysis. The subjective parameters used were mental demand, physical demand, effort, frustration, and self-perceived performance. The participants also rated the intuitiveness of the arm support, which was solely used for an indication and not for comparisons. Position data for the arm cups were derived from rotation sensors on the arm supports during trials that involved arm support.

### Data analysis & interpretation

Data collected from all participants were analyzed with IBM SPSS (version 28.0.1.1 (15), SPSS, Inc., Chicago IL, USA). The normality of the data were tested using the Shapiro-Wilks test. Paired t-tests were used if the data were normally distributed, while the Wilcoxon Signed Rank test was used with non-normally dis- tributed data. The two datasets (with and without support) were compared within each trial to identify differences in performance. Also, the first and last trials of the individual datasets were compared to find learning effects. Differences were determined significant for *p* < 0.05. The data of the questionnaire were analyzed using the same method as the performance data, to spot differences in the experience of participants on the AdLap-VR, with and without the arm support.

## Results

### Design

The prototype met all the requirements. The system consisted of multiple linkages, providing four degrees of freedom at the arm pad. Rotational joints in the horizontal plane create translation along the x and y axes and rotation around the z-axis, while the four-bar mechanism is responsible for translation along the z-axis. All the linkages were constructed using aluminum square rods, and the joints consisted of ball bearings in combination with solid steel rods. All the custom-made parts at the joints were fabricated from aluminum using a milling machine.

**Fig. 3:**
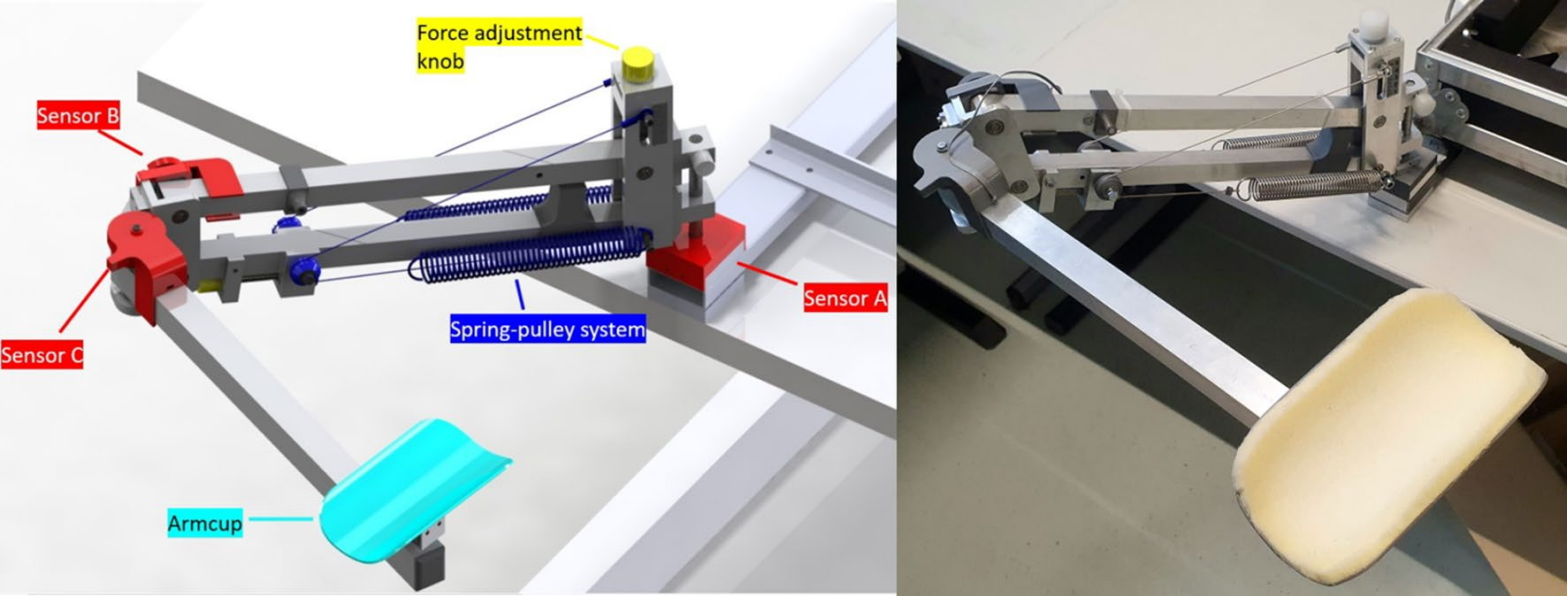
Dynamic Arm Support 3D-design (Left) and prototype (Right)

The primary design requirement was to support users’ arms throughout the entire range of motion necessary for using the AdLap-VR. In the conceptual phase, it was determined that steel springs would be used to achieve this goal due to their durability and simplicity of use. A balanced system with adjustable support force based on a “zero-free length spring” design, as described in by Herder et al., on balancing mechanisms was integrated in the design [12, 13]. This design combines the increase in strength of the spring with a decreasing effective pulling angle on the bottom rod of the four-bar mechanism, canceling each other out when lowering the support height. This results in a mechanism with a constant support force in the working range, as opposed to a variable support force. The mechanical principle and functional parts are shown in Fig. 3. It is a requirement that the spring depicted in Fig. 3 can only be stretched and never compressed, which was achieved through the use of cables and pulleys symmetrically attached.

The support force is adjustable by rotating a knob on top of the support’s base, which, in turn, pulls the attachment point of the cable upwards via a spindle. As the height of the attachment point increases, the moment exerted on the bottom rod increases, leading to an increases in the support force. Further details on the sensor development and functioning used to tract the arm support’s movements can be found in supplemental file A3.

### Pilot study

**Fig. 4:**
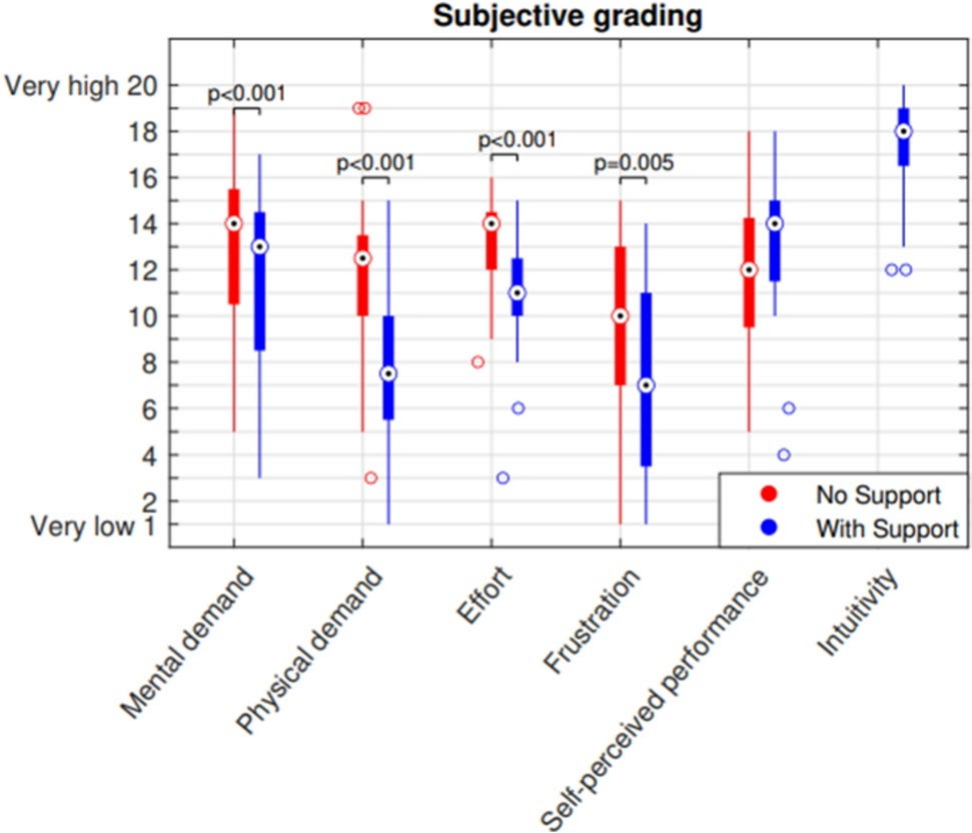
Boxplots of the questionnaire results

A total of 20 students participated in the study, resulting in 160 performed trials on the AdLap-RS. All participants completed the pre-tests. Two participants exceeded the 5-min time limit in the pre-tests, and none did so during the trials. Only one participant could not complete the second part of the trials due to external circumstances and had to be excluded from the analysis. Therefore, a replacement participant was recruited. All participants completed the questionnaire. Participants found the task, in combination with the arm support, significantly less mentally and physically demanding, requiring less effort, and resulting in less frustration. All participants expressed a high score for the intuitiveness of the device (Fig. 4).

**Fig. 5:**
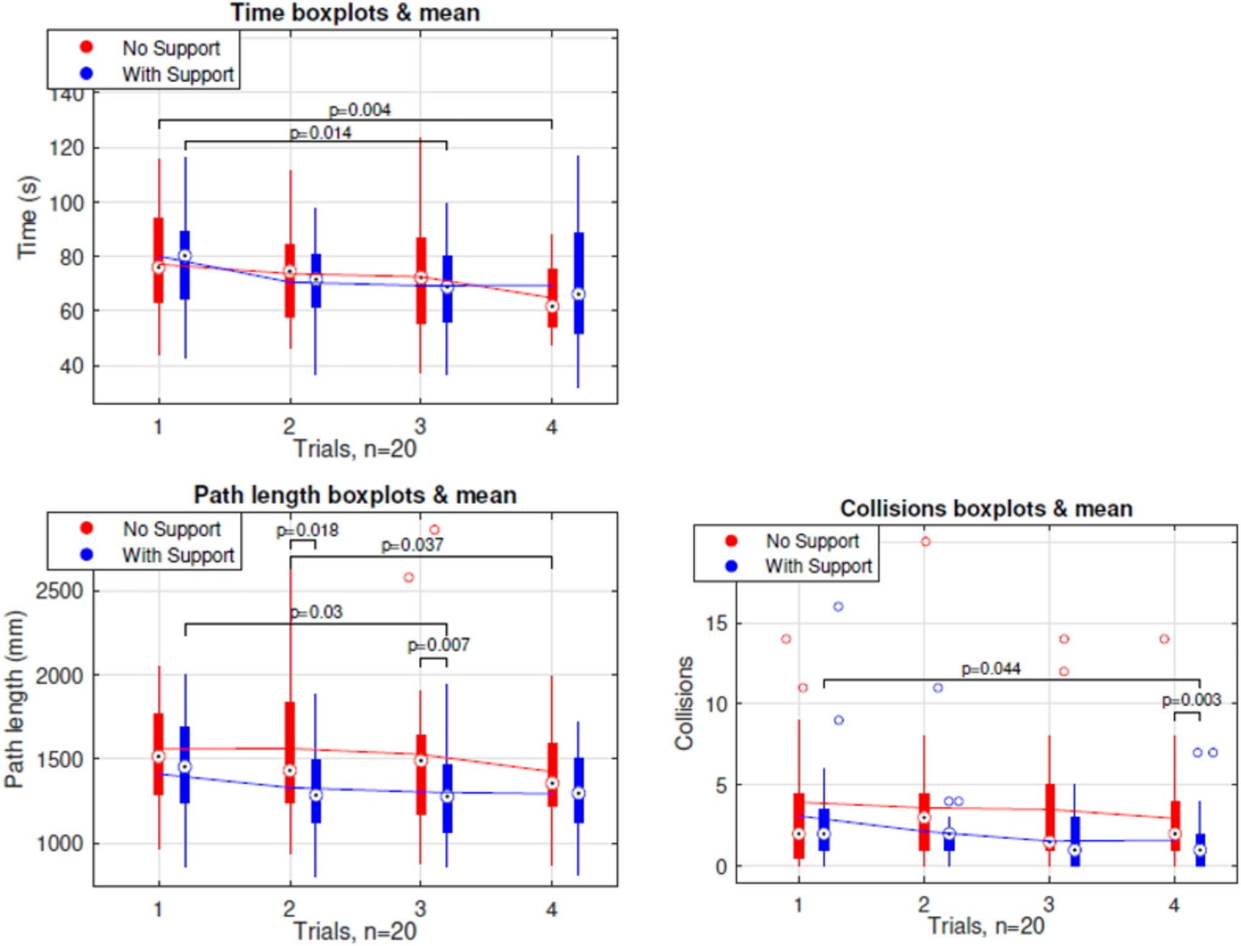
Boxplots of the AdLap-VR performance parameters task time, path length and collisions per trial number

Between the first and last trials of the task without arm support, there was a significant decrease in time, as well as between trials 1 and 3 of the task with arm support (Fig. 5).

**Fig. 6:**
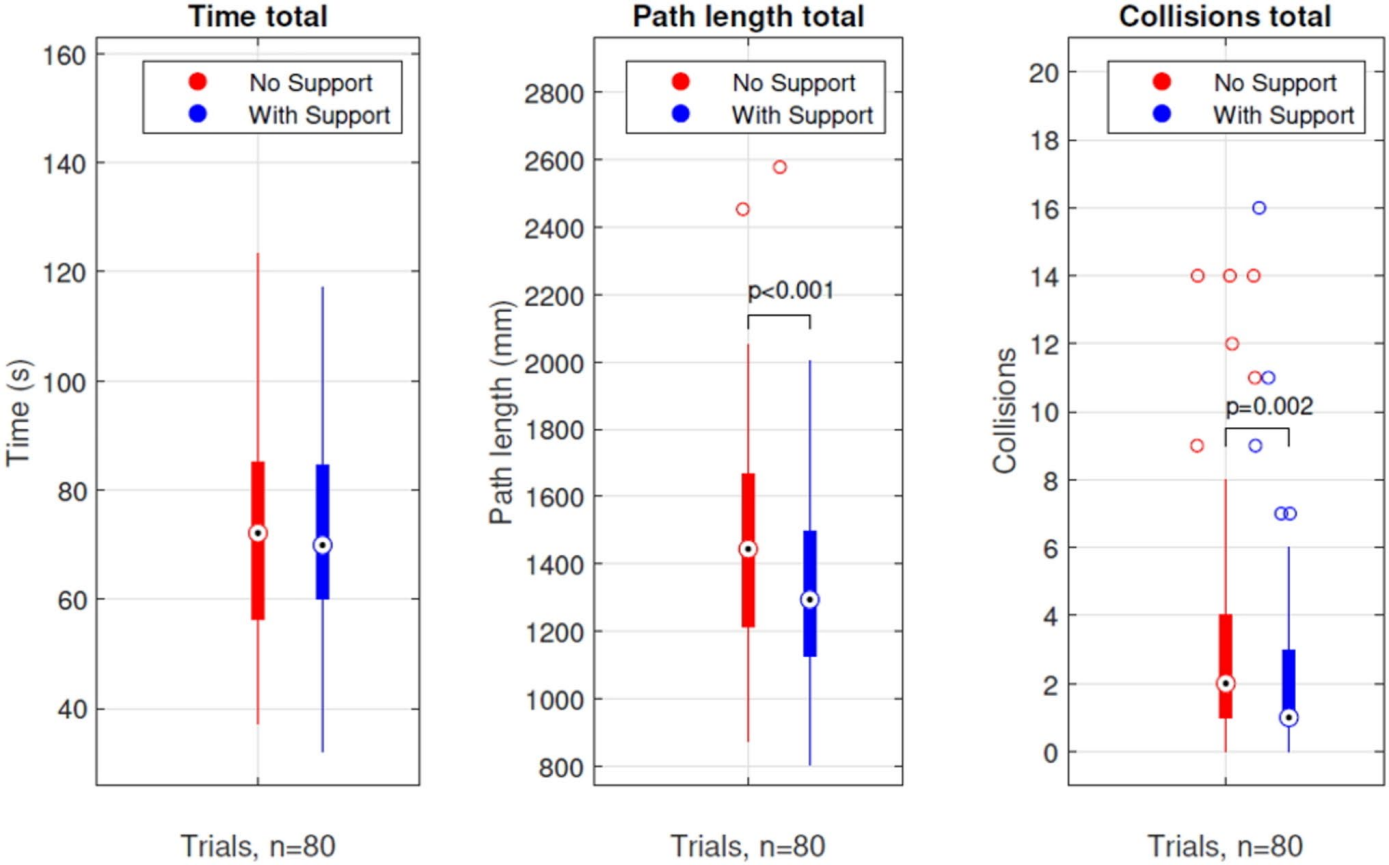
Boxplots of the AdLap-VR performance parameters task time, path length and collisions of all trials combined

Regarding the collision data, a significant decrease in the number of collisions was observed between trials 1 and 4. In trials 2 and 3, participants had significantly lower path lengths when using arm support compared to trials without arm support. In the final trials, participants also had significantly fewer collisions when using compared to trials without arm support. Figure 6, shows that the participants, overall, had significantly lower path length and fewer collisions in the tasks with the novel arm support as compared to having no arm support.

**Fig. 7:**
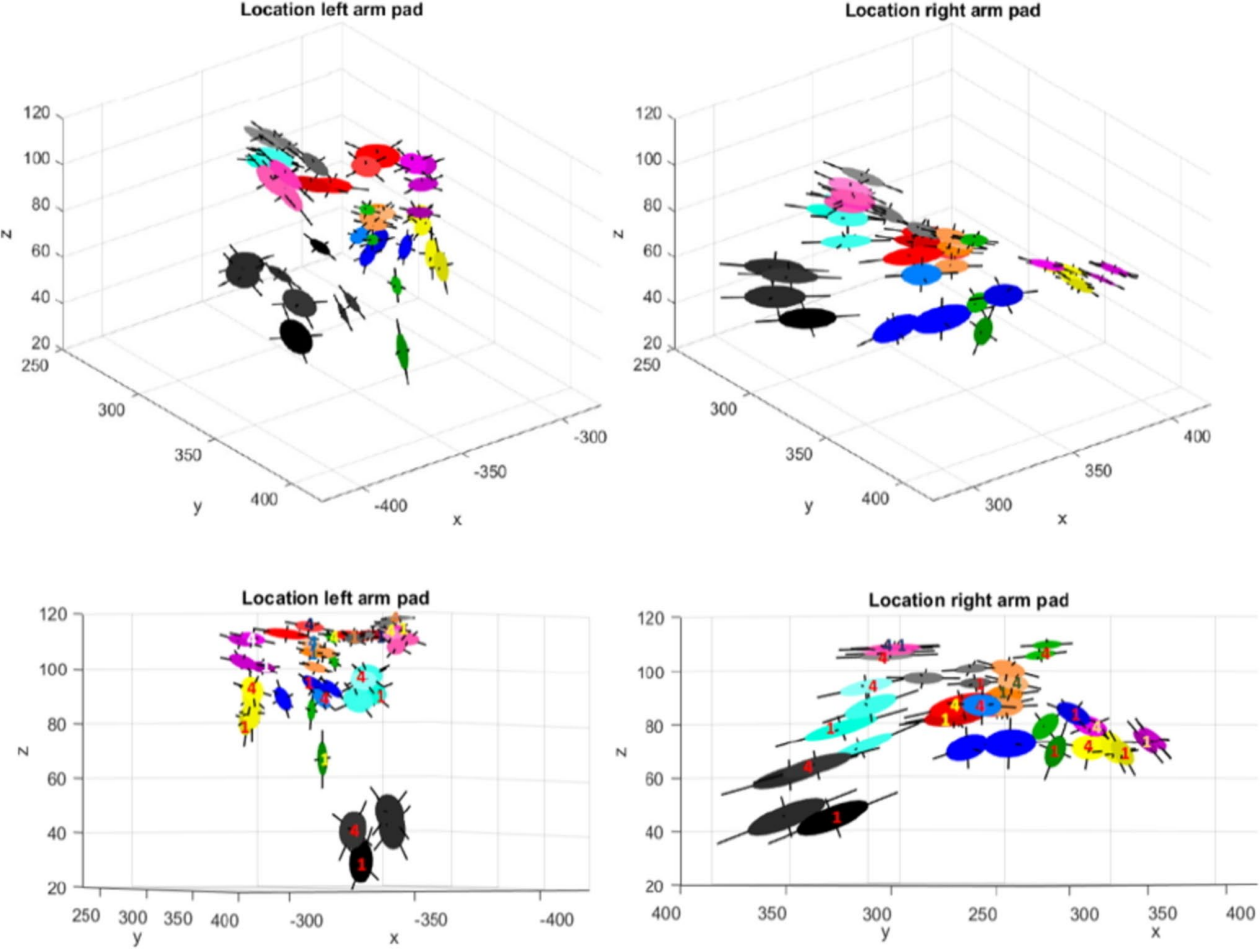
3D Graphic display of the positional areas of the forearm pads of the participants in trials 1 to 4. Participants are distinguished by color

The movement during the arm support of all recorded trials is shown in Fig. 7. To prevent data cluttering, only the first ten participants are displayed. The variability of movement and the main directions are graphically presented as projections of oriented ellipsoids with the principal axes extending from the surface. The data shows that for the individual participant, the position of the handle in space migrate during the training task, the principal components do not change much. Between the participants position differences up to 800 mm in the horizontal plane and 700 mm in the vertical plane were observed.

## Discussion

A novel adjustable passive dynamic arm support was designed, produced and validated, meeting all design requirements and delivering reliable and repeatable readings. The arm support effectively balanced the arms of all users during tasks on the AdLap-VR robotic laparoscopic simulator. The results of the study demonstrate increased perceived comfort in multiple aspects during tasks, along with improved objective performance for the parameters path length and collisions. The prototype operated smoothly as expected, requiring no maintenance. Feedback from the users consistently indicated that the dynamic arm support provided a more relaxing experience.

### Performance data

Although learning effects can be observed in Fig. 5 for the three different parameters, it appears that the curve for task time shows almost no differences between the two conditions, suggesting that the presence of an arm support does not add complexity to the task execution. For path length and collisions, the parallel learning curves have a somewhat similar profile but with an offset. Significantly lower path length and collision rates were observed for the combined trials with arm support compared to the combined trials without arm support, whereas the parameter of time showed no significant differences (Fig. 6). A shorter path length with equal time indicates a reduction in participants in the average speed of the participants on the AdLap-RS [10, 14]. This reduction in speed could explain the lower number of collisions, as it suggests slower, more stable, and more precise movements. This data aligns with the majority of participants’ statements after their experience with the arm support. Moreover, in trials without arm support, participants tend to position their non-working arm towards a resting position, lowering the upper arm parallel to the upper body to reduce shoulder effort. This positional change from the working position to the resting position and vice versa creates extra path length. In contrast, during trials with arm support, participants maintain their non-working arm in a position similar to the working position, as the arm support prevents fatigue. This likely contributed to a decrease in path length for participants in trials with arm support.

### Posture

Figure 7 shows that the movement areas are grouped per participant, indicating only minor adjustments in posture as participants learned and aimed to improve their performance. These adjustments were limited as participants were bound by the unchanging dimensions of their body, likely related to the height of the equilibrium point of the system arms (i.e., balancer and master arms combined). In the vertical plane (i.e., z-axis), motions were more pronounced compared to the horizontal plane, potentially due to small changes in siting position. Regarding the vertical plane, eight out of ten participants increased the height of their left arm, and nine out of ten participants increased the height of their right arm between the first and last trial with arm support (Fig. 7). This change in arm height can be explained by a development in strategy of the participants for the specific task. It was observed that grabbing the marbles from the top as sort of a crane with a grab is a more efficient way of performing the task than grabbing the marbles from the sides. Most participants intuitively learn this and moved toward this better strategy during the trials. This can explain the trend of the increasing height of the arm positions of most participants, as the more optimized strategy requires a higher position of the AdLap-VR handles, and thus a higher position of the arm supports for comfort. Another interesting observation is that the size of the ellipsoids in Fig. 7 of each participant, and the main directions of variability from the principal axes, appear to stay the same throughout the trials but are different between participants. This indicates that participants keep the same dimensional movement patterns but express them from different starting locations throughout the trials, due to slightly different postures.

When reviewing Fig. 7, the black, green, and yellow ellipsoids appear significantly lower placed than the remainder of the ellipsoids. However, upon further inspection of the data, no further conclusions can be drawn from the baseline demographics. One cause could be that both participants set the arm support strength at an insufficient level, causing the arm to find a supporting equilibrium height lower than other participants. Finally, the large operating differences between participants in general indicates the importance of dynamic arm support over a static arm rest. This makes sense as the level and location of support should not be defined by the console but by differences in body and extremity dimensions, variations in muscle strength and length of the procedure. Moreover, surgeons should be able to reposition their arms in order to prevent fatigue and overloading during the procedure.

### Limitations

Firstly, the system was only tested with students, not actual practicing surgeons. While this study serves as a good indicator of the design’s functionality, it is still uncertain how the arm support improves the experience of surgeons within the operating room. Further research, incorporating practicing surgeons as participants, is needed to determine the clinical utility and impact of the arm support on behavior (e.g., clutching of the handle vs instruments), surgery outcomes and ergonomics [15]. Secondly the trial times for the short marble sorting task are not representative of the extended durations of surgical operations that can last for hours. Some participants from the presented study even noted that the short trial times did not sufficiently induce fatigue, thereby limiting their ability to discern differences in comfort levels. This most likely limited differences in performance as well, as a paper by Z. Tsafrir [7] on laparoscopic performance describes that the time to complete tasks increases with increased tiredness. This effect was not found in the current study, where the parameter time showed no differences between groups. To fully investigate the impact of the designed interventions on the total comfort experience of surgeons, trial times can be increased in future studies to allow for the onset of fatigue, enhancing the applicability of the findings to real-world surgical scenarios.

### Design recommendation

To further develop the system, to automate the procedure of adjusting the support strength to the arm weight of the user with the use of force sensors, as some participants indicated that they found it difficult to find the right setting. This could be achieved by replacing the turning knob on top of the system with a small motor.

## Conclusion

A novel passive dynamic arm support was designed, developed and validated that has been shown to increase comfort while also enhancing performance on tasks using the AdLap-RS robotic surgery skills simulator, likely due to increased stability. The preliminary results provide a useful indication of the functionality and effectiveness of the novel-designed arm support for its use in RALS and should help with improving the ergonomics in the next generation of Robotic surgery. Going forward, the arm support must be assessed in a clinical setting to examine its utility and effect on surgery outcomes.

## Supplementary Information

Below is the link to the electronic supplementary material.

Supplementary file1 (DOCX 4664 KB)

Supplementary file2 (DOCX 1242 KB)

Supplementary file3 (DOCX 294 KB)

## Data Availability

Upon publication, the data will be published on the 4tu researchdata repository of the Delft University of Technology at https://data.4tu.nl/.
