# Supplementary material for: Design and evaluation of a smart passive dynamic arm support for robotic-assisted laparoscopic surgery
Source: J Robot Surg. 2024 Feb 10;18(1):71. doi: 10.1007/s11701-024-01820-1 (PMC10858817; doi:10.1007/s11701-024-01820-1)

**Supplemental File 1 Literature review**

# Introduction

Surgeons performing robotic assisted laparoscopic surgery experience physical stress and overuse of shoulder muscles due to sub-optimal arm support during surgery. In an attempt to develop an improved arm support system, background research is necessary into the topic of passive dynamic arm supports. The objective of this literature study is to present a systematic review of all passive dynamic arm supports found in the literature, classifying them into distinct working principles and presenting the most promising working principles for the applications within robotic assisted laparoscopic surgery. The PRISMA method was used to conduct the literature review, using three search engines: PubMed, Scopus and WebOfScience. The articles went through an identification phase, screening phase, eligibility phase and the inclusion phase. The arm supports of the included articles were analysed for their working principle and different characteristics. 68 Papers were included in this report, resulting in 74 unique arm supports. The arm supports could be classified into the following working principles: single pivot point mechanisms (2,6%), single linkage mechanisms (4%), multiple linkage mechanisms (12%), direct pulling cable mechanisms (13,3%), 4-bar mechanisms with the base as a vertical linkage (30,7%), 4-bar mechanisms without the base as a vertical linkage (4%), torsion around shoulder based mechanisms (16%), spring-loaded lever mechanisms (13,3%), leaf spring mechanisms (4%) and mechanisms with multiple springs acting as shoulder muscles (1,3%). It is estimated that 4-bar mechanisms with an added lever, are the most promising mechanisms for the applications within robotic assisted laparoscopic surgery. This report can serve as the first step in developing a new arm support for the robotic master interfaces.

# Results

In the results, the arm supports are shown with their working principles, classified into distinct categories and judged on multiple characteristics.

## Categories of mechanisms based on rotation only

A few dynamic arm supports were found that only allow rotation without any translation. This is rotation around two axes, so the user has two DoF ([Figure 1](#_bookmark0)). It is rarely mentioned in the literature, probably because of its unpractical use: to grab something from down low, the user has to move his elbow very high. Also, this type of arm support lacks a fundamental range of motion for most uses, the translation.


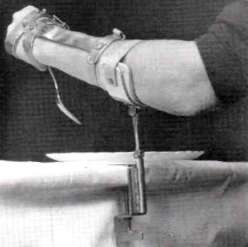


Figure 1: C-Clamp, an arm support rotating around two axes

## Categories of mechanisms that allow xy-translation

This subsection of supports are supports that are based on linkages with rotational joints with the rotational axes parallel to the z-axis. Therefore, they only allow movement in the xy plane, supporting the arm in the z-direction on a fixed height. They consist of

- A single linkage, [Figure 2](#_bookmark1)
- Multiple linkages, [Figure 3](#_bookmark2)

These type of supports are used in rehabilitation, as well as everyday desk work such as mousing behind a computer. They can be height adjustable as well.

## Categories of mechanisms that allow xyz translation

### Direct cable pulling mechanisms

Cable pulling mechanisms pull the arm(s) of the user from a vertical cable, leaving the arm in a stable hanging position. The cable is balanced in three ways:


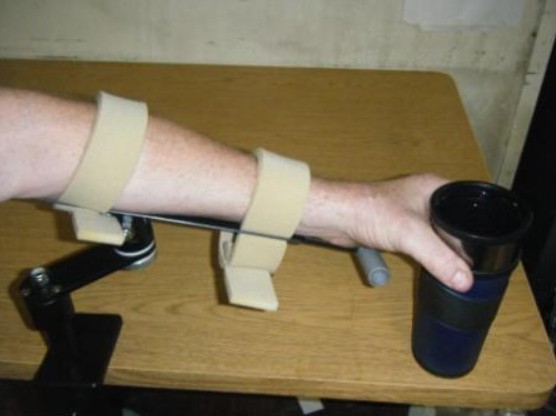


Figure 2: ’Thomas steady arm’, a single-linkage xy-plane arm support


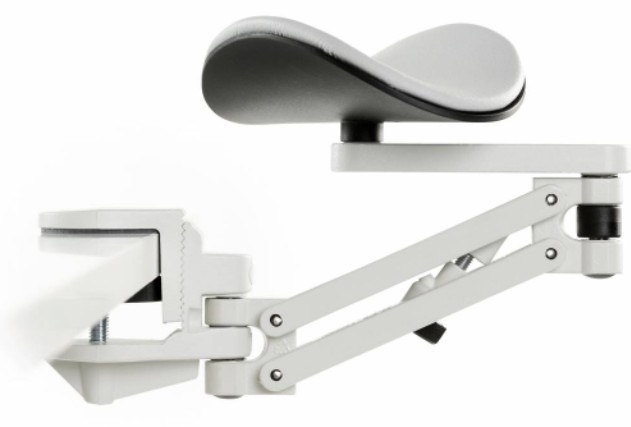


Figure 3: The Ergorest, a multi-linkage, height adjustable, xy-plane arm support

- - - - Mass balanced
      - Spring balanced
      - User balanced

They are mostly used for people with decreased arm function due to the usually large size and overhead nature of the mechanisms.

### Mass balanced

In a mass balanced cable, a lever arm mechanism is made with the weight of the arm on one end and a counterweight on the other end. The counterweight creates a moment around the rotational joint in the middle of the rod, which balances for the moment that is created by the weight of the arm of the user on the other end of the rod. This can be seen in [Figure 4](#_bookmark3).


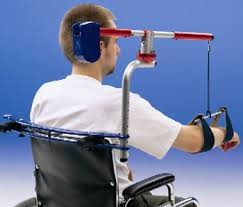


Figure 4: The Nitzbon Mobility Arm, a mass-balanced cable pulling mechanism

### Spring balanced

In spring balanced mechanisms, the tension on the cable is countered by a spring or a mechanism with springs, an example is seen in [Figure 5](#_bookmark4).

### User balanced

User-balanced mechanisms require the user to provide the tension on the cable for lifting the arm. This is done by extending one arm, which lifts the other arm up. Only one arm can be lifted at a time ([Figure 6](#_bookmark5)).

### 4-bar mechanisms with the base as a vertical linkage

The group 4-bar mechanisms with the base as a vertical linkage is sectioned into the following groups and subgroups:

- - - - Single 4-bar

**–** With added lever


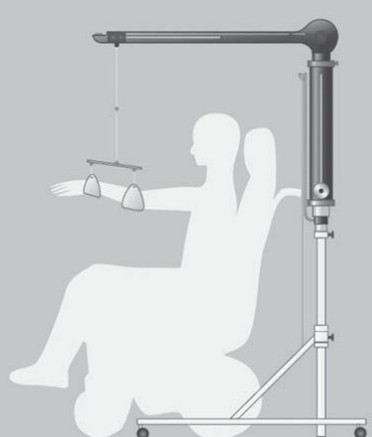


Figure 5: ’The Sling’ from Focal Meditech, a spring-balanced cable pulling mechanism


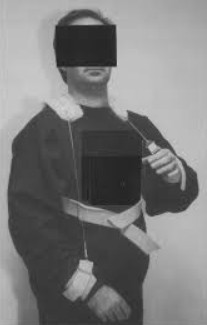


Figure 6: Dynamic triceps driven orthosis (DTDO), where one arm pulls the other arm upwards with a cable

- - - - Double 4-bar

They make use of a reference frame, which can be the users body or a wheelchair for one of the links.

### Single 4-bar mechanisms

Single 4-bar arm supports use the link parallel to the ’base’ link for attaching arm cups or a linkage to the arm cup for extra DoF. The parallel link of the 4-bar usually stays vertical throughout its use, such as in [Figure 7](#_bookmark6)


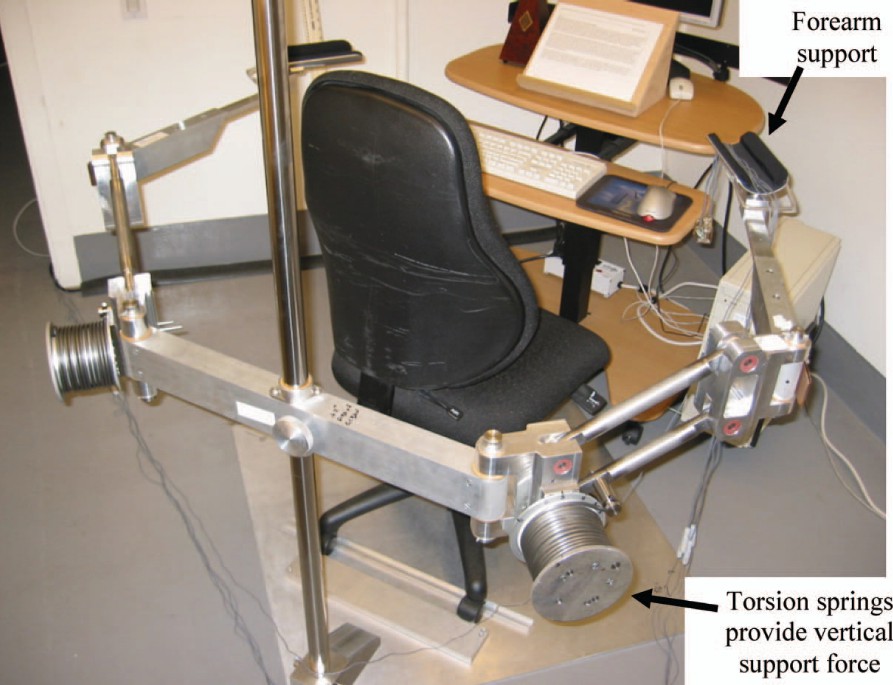


Figure 7: Prototype of an arm support with a 4-bar mechanism with an extra linkage for an added DoF

### 4-bar with added lever

4-Bar mechanisms with an added lever are typically used for when the system provides support at two locations. This could be the upper- and lower arm, but also the elbow and wrist. The 4-bar part acts as the main support of both the upper and lower arm and provides a stable vertical basis for the lever, as it always stays perpendicular to the ground. An example is seen in [Figure 8](#_bookmark7).

### Double 4-bar mechanisms

Double 4-bar mechanisms have the same characteristics of single ones, but they have the two 4-bars linked to each


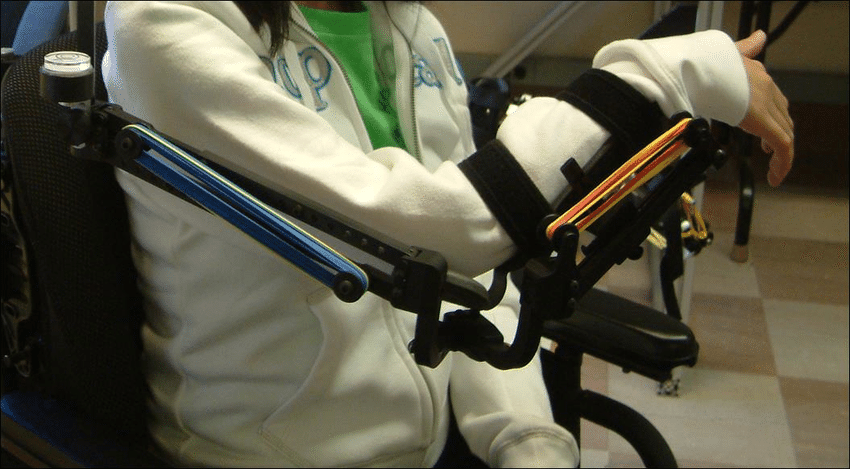


Figure 8: ’The Wrex’, an arm support with a mechanism consisting of a 4-bar with an added spring-loaded lever

other by combining one of their vertical links as a rotational joint around the z-axis. This allows significantly more range of motion than single 4-bar mechanisms. An example is seen in [Figure 9](#_bookmark8).


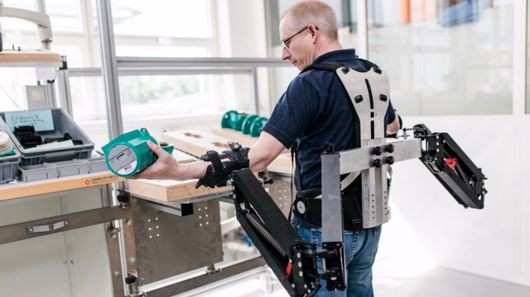


Figure 9: ’Robo-Mate’, an exoskeleton arm support using two linked 4-bar mechanisms

### 4-bar mechanisms without vertical linkage as a base

This is a seperate group of systems, as the 4-bar mechanism of these arm supports are only supported in one rotational joint, while the previous discussed arm supports have the base acting as the vertical linkage. An example can be seen in [Figure 10](#_bookmark9).


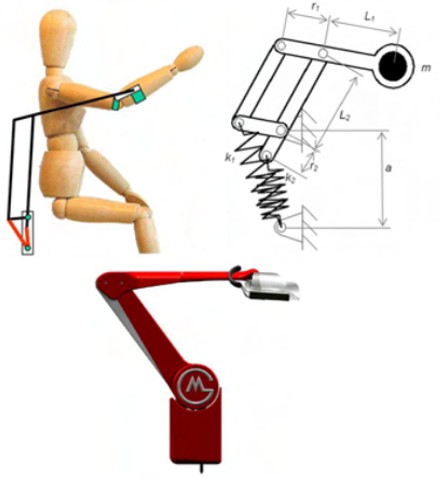


Figure 10: ’ARMON’, a 4-bar mechanism arm support without the base as a vertical link

### Mechanisms with a single lever with torsion

These type of arm support systems mostly make use of a mechanism inside of a ’black box’ around a single joint to generate torsion, such as [Figure 11](#_bookmark10). This joint is usually located at the shoulder of the user. For the instances that

the black box is shown in articles, it houses multiple parallel springs in combination with a lever gear, which can be seen in [Figure 12](#_bookmark11), as well as in [[1](#_bookmark19)]. 9 Out of 11 of the passive exoskeletons fit into this category. Even though the full working principle inside the black box is not always clear, this category is based on the fact that the torsion is generated around a single joint onto a lever that supports the arm.


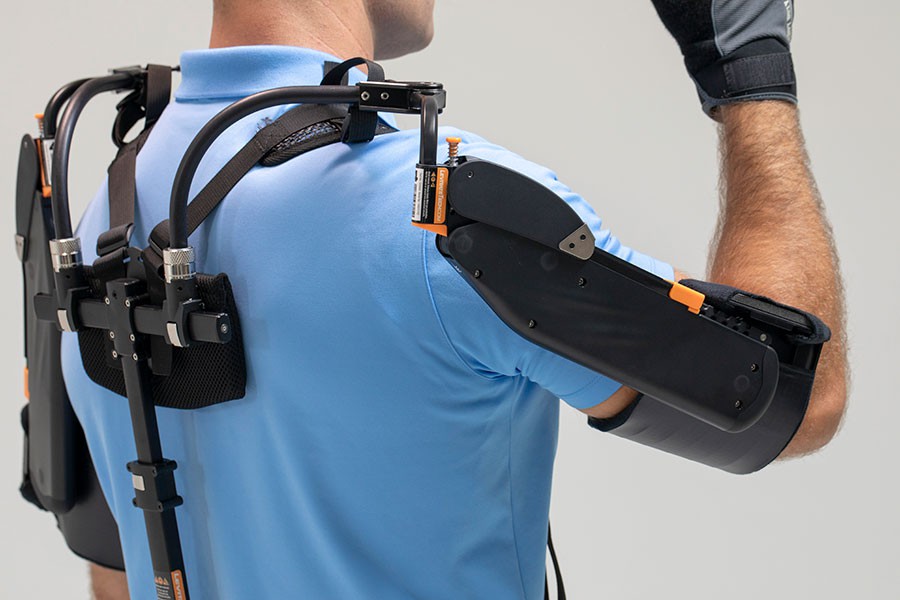

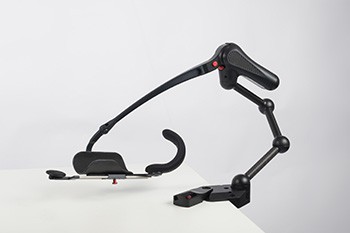


Figure 11: Examples of arm supports with mechanisms actuated by torsional forces around a single axis of the lever that supports the arm. Left: Exoskeleton ’Levitate’ right: ’Dowing’


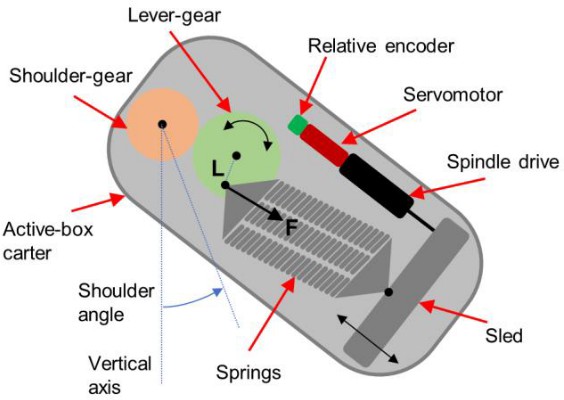


Figure 12: The inside of a black box functioning as a torsional spring. This one has a servomotor for motorized tuning of the torsional force [**?**]

### Lever mechanisms with extension springs

Spring loaded lever mechanisms are mechanisms where a spring-loaded lever with an arm cup is attached to the arm of the user.

### Single lever

Single lever mechanisms have a lever with a rotation point at the shoulder, which is then actuated by an extension spring or band, such as in [Figure 13](#_bookmark12).


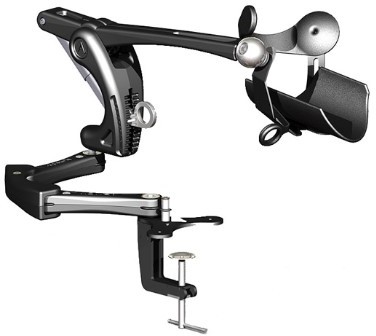


Figure 13: ’Armon Edero’, a single lever arm support actuated by an adjustable extension spring.

### Double lever

Double lever mechanisms use two levers to support the arm in two places, mostly the upper arm and the lower arm. The levers are actuated by extension springs or bands, or strings on pulleys attached to springs. This type of mechanism is heavily dependant on proper dimensioning of the spring attachments to work and can become fairly complicated. It can be seen in [Figure 14](#_bookmark13).


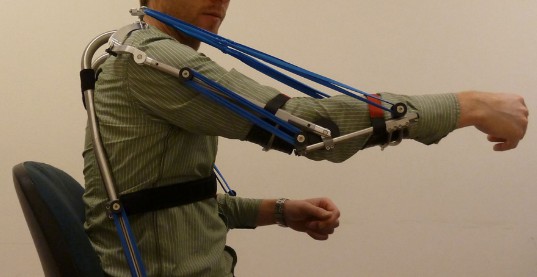


Figure 14: ’A-gear’, a double-lever arm support mechanism supporting the arm in two places [[2](#_bookmark20)].

### Leaf spring mechanisms

These type of mechanisms differ significantly from each other but use leaf springs as their form of passive actuation. It is an interesting type of mechanism, as leaf springs are able to store energy while still being close to the body and can act in structural ways as well. Skelex uses their leaf springs in an advanced way, combining two tasks: they store energy by bending and function as the main structural component of the exosuit by transferring the weight of the arms into the waist ([Figure 15](#_bookmark14)), while other exos need an additional solid piece of metal from the waist to the shoulders for this.


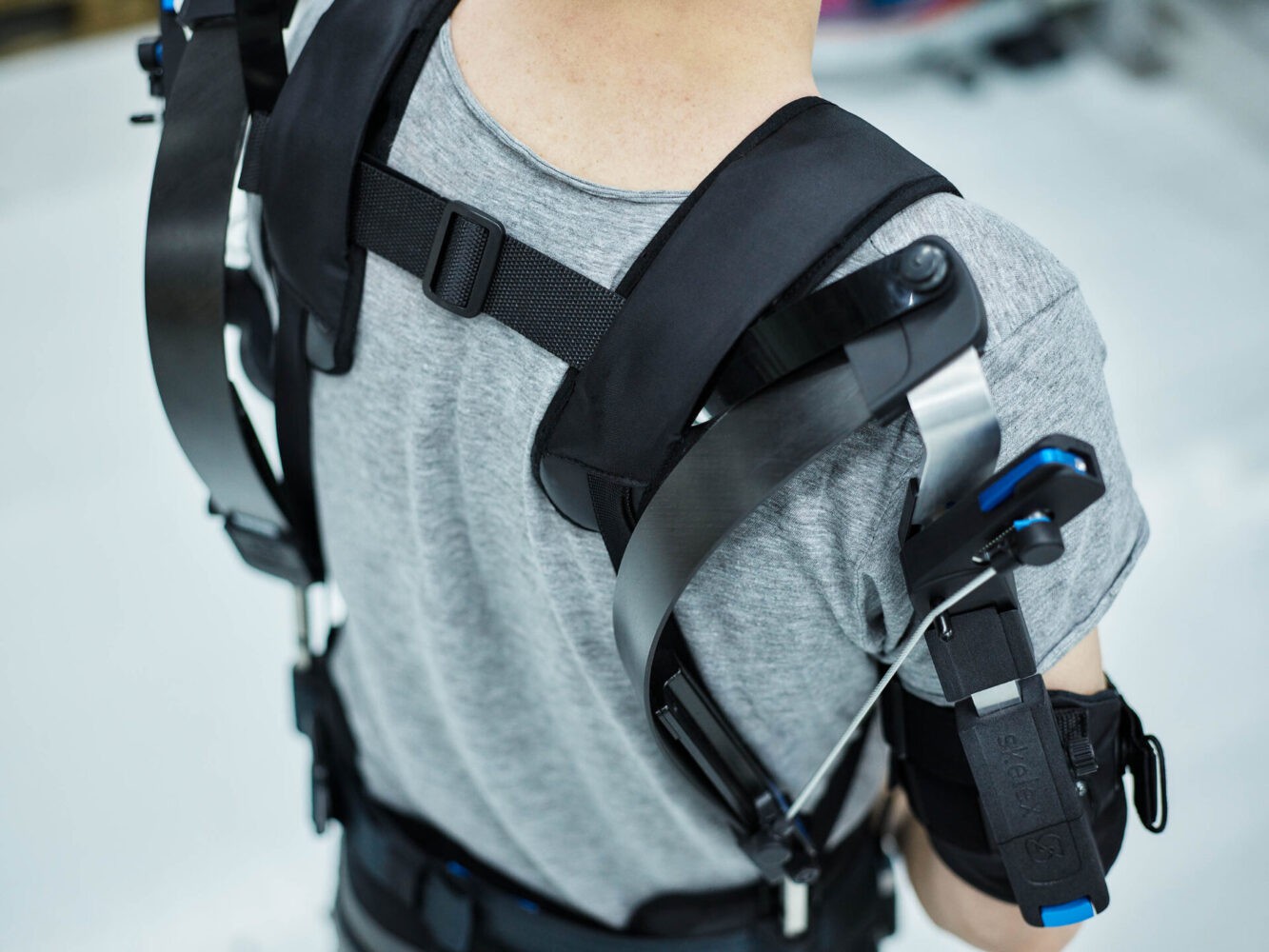


Figure 15: ’Skelex-360’, an exosuit with bending beams acting as actuators and structural components. .


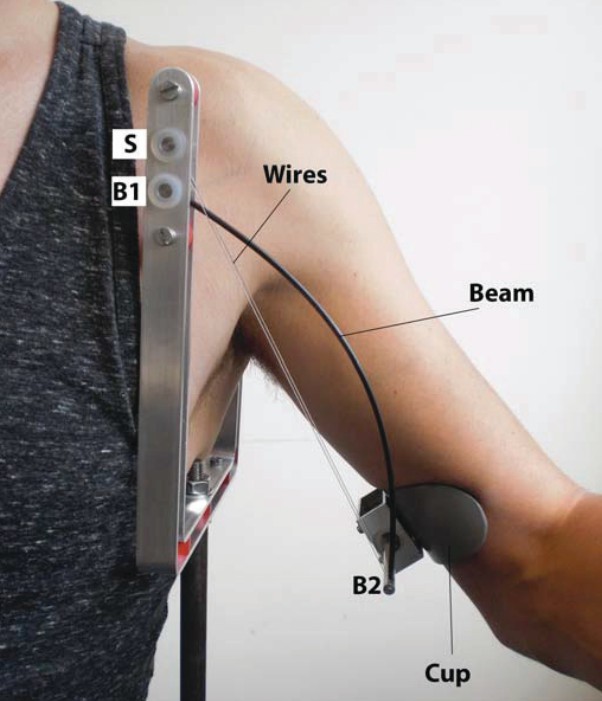


Figure 16: Prototype of an arm support where bending beams function as the passive actuator. The beams support the arm in gravity but also counter rotation of the arm.

### Multiple springs as shoulder muscles

The last proposed group of systems comes from an exoskeleton that has springs attached between the elbow and the shoulder. As the springs aligned with the direction of the shoulder muscle fibers, it is expected to aid this muscle in lifting the arm and thus reduce strain on the shoulders, mainly in abduction but also some in flexion. [Figure 17](#_bookmark15)

## Proposed Classification tree [Figure 18](#_bookmark16)

The arm supports where classified into 3 distinct categories, based upon the movement planes. Allowing rotation only, allowing rotation and xy translation, and supports that allow xyz translation. This was done because the first


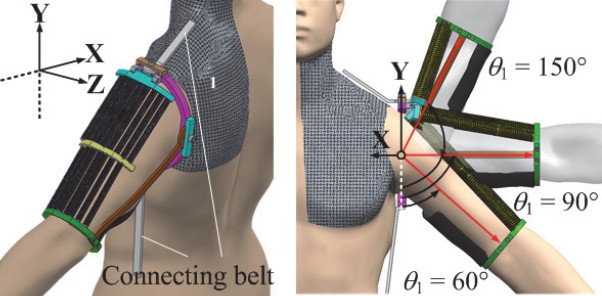


Figure 17: Unique mechanism with springs in the direction of the shoulder muscle

two categories lack fundamental support for vertical movement patterns of the user. The second stage of the classification was based on the working principles of the arm supports, with each category having its own color for clarity. Some of these categories have subcategories. In the last stage of the classification, the arm supports are assigned to the design phase they were in according to the articles.


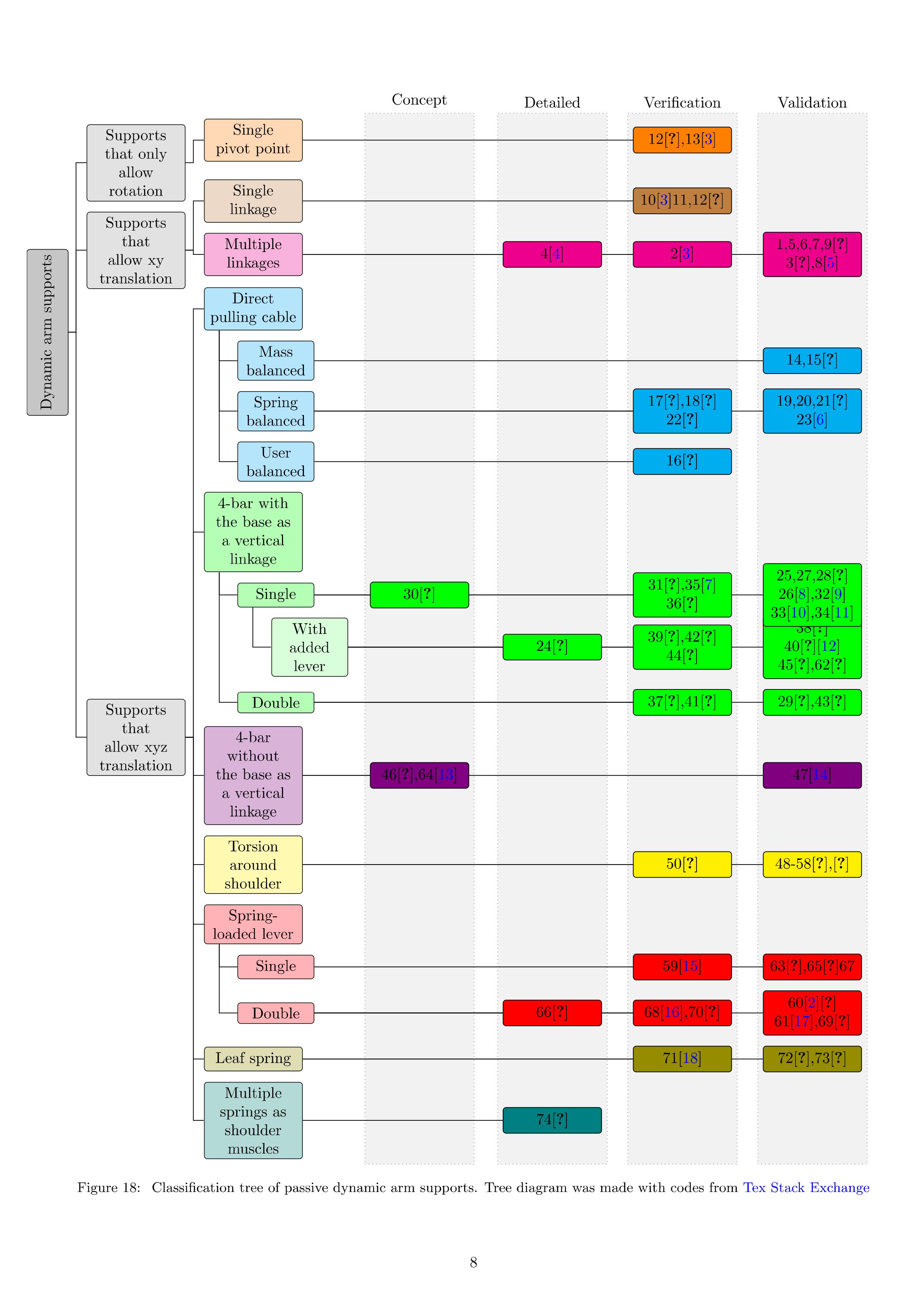


## Distribution of papers throughout the years

In [Figure 19](#_bookmark17) we can see the amount of included papers per year in a graph. A small number of published papers in the early 2000’s can be seen, as well as a sudden spike in 2007. Following 2011, a steady increase is seen which drops back down after 2016. Then 2020 and 2021 both had a substantial amount of papers.

8

7

6

Number of used papers

5

4

3

2

1

0

1997

1998

1999

2000

2001

2002

2003

2004

2005

2006

2007

2008

2009

2010

2011

2012

2013

2014

2015

2016

2017

2018

2019

2020

2021

Publication year

Figure 19: Histogram of the number of used papers each year for this literature review

## Arm supports characteristics

The arm supports were analyzed and rated on multiple characteristics. These were:

- Mounting type
- Location(s) of support
- Volume
- Range of Motion
- Complexity
- Working principle

### Mounting type

The mounting type could be analyzed from the pictures shown in the articles. These were the four mounting methods seen:

- Body worn (31,0%)
- Immovable Object (18,9%), such as a table or chair
- Movable object (39,2%), such as a wheelchair
- On a base (10,8%), so a stand designed specifically for the arm support to act as the stable base

### Location(s) of support

Most of these could also be seen from the pictures in the articles. Two articles only showed concept drawings where a clear location for the support was not yet determined. The location(s) were:

- Elbow & forearm (25,7%)
- Upper arm (23,0%)
- Forearm (24,3%)
- Upper arm & forearm (9,5%)
- Forearm & wrist (5,4%)
- Upper arm & wrist (1,4%)

- Elbow (1,4%)

- Wrist (2.7%)

- Upper arm, forearm & wrist (4,1%)
- Unknown (2,7%)

3


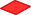

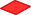

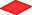

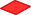

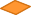

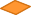

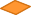

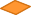

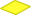

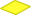

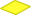

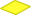

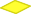

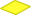

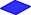

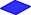

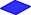


A

B

C

D

E

Average complexity

2

1

0

Body worn

RoM level F

Immovable object

Moveable object

One a base

Category

Figure 20: 3D Histogram of the mounting types of arm supports in different range of motion levels with their associated average complexity level.

F

E

Average RoM Level

D

C

B

A

Elbow & forearm

Upper arm

Forearm

Upper arm & Forearm

Forearm & wrist

Upper arm & wrist

Elbow

wrist

Wrist

Unknown

Location(s) of support

Upper arm, forearm

Figure 21: Histogram of the average range of motion level per location(s) of support. RoM levels where given a number score from A=1 to F=6, then combined and their average was calculated.

### Volume

The volume was judged with a score ranging from very small to very large, relatively to each other. No objective scores were used as this category was more used to compare the arm supports. The levels turned out to be:

- Very small (1,4%)

- Small (25,7%)

- Medium (43,2%)

- Large (25,7%)

- Very large (4,1%)

The ”very small” score was given to the arm support with the least volume: an arm support fitted into the clothing of the user, PlaySkinLift. ”Very large” was given to the arm support with the most volume: A two meter tall pulling cable mechanism with a base, Freeball. The other scores where based relatively to these.

Very large

Large

Average Volume

Medium

Small

Very small

Body worn

On a base

Mounting type

Immovable object

Movable object

Figure 22: Histogram of the Average volume for arm supports with different mounting types

### Proposed level classification of the Range of Motion

For the range of motion, the translation was most important, as all of the arm supports allowed wrist movement and therefore flexion, extension, radial deviation and ulnar deviation. They also all allowed elbow extension and flexion. Thus, the difference between the range of motion was found in the planes where movement was allowed, and the reach within those planes. The amount of translation (small, medium, large) was judged relatively to one another.

The information was extracted from making an estimation based on the pictures in the articles and, if necessary, from online videos.

- Level A: All (20,3%)
- Level B: All xy-translation, large z-translation (14,9%) - movements like painting
- Level C: All xy-translation, medium z-translation (40,5%) - movements like bringing a spoon to the mouth
- Level D: Medium xy-translation, small z-translation (6,8%) - movements like writing, petting a cat
- Level E: Medium xy-translation + rotation (14,9%) - movements like using a computer mouse
- Level F: Rotation only (2,7%)

### Complexity

There are diverging perspectives in the literature on how to measure the complexity of a certain design. The decision was made to measure complexity based on how much a certain system could be decomposed into smaller systems/parts, as this method is used to assess system difficulty and compare systems [**?**]. Complexity is therefore judged as follows:

- Low: a maximum of 3 moving parts (47,3%)
- Medium: 4-8 moving parts (36,5%)
- High: 9 or more moving parts (16,2%)

The final overview of all the systems with all their characteristics is shown in [Appendix A](#_bookmark37).

In [Figure 23](#_bookmark18) a 3D-histogram was made to show the connection between the working principles and their associated range of motion level and the number of systems that fit those categories.

15


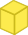

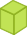

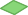

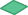

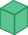

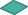

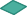

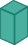

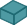

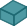

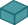

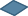


A

B

D

C

E

Amount of systems

10

5

0

Single pivot point

Single linkage

RoM level F

Multiple linkages

Direct pulling cable

4-Bar with vertical linkage

4-Bar without vertical linkage

Torsion around shoulder

Spring-loaded lever

Leaf spring

Multiple muscle springs

Category

Figure 23: 3D Histogram of the category of systems vs the range of motion and the number of systems that fit the category


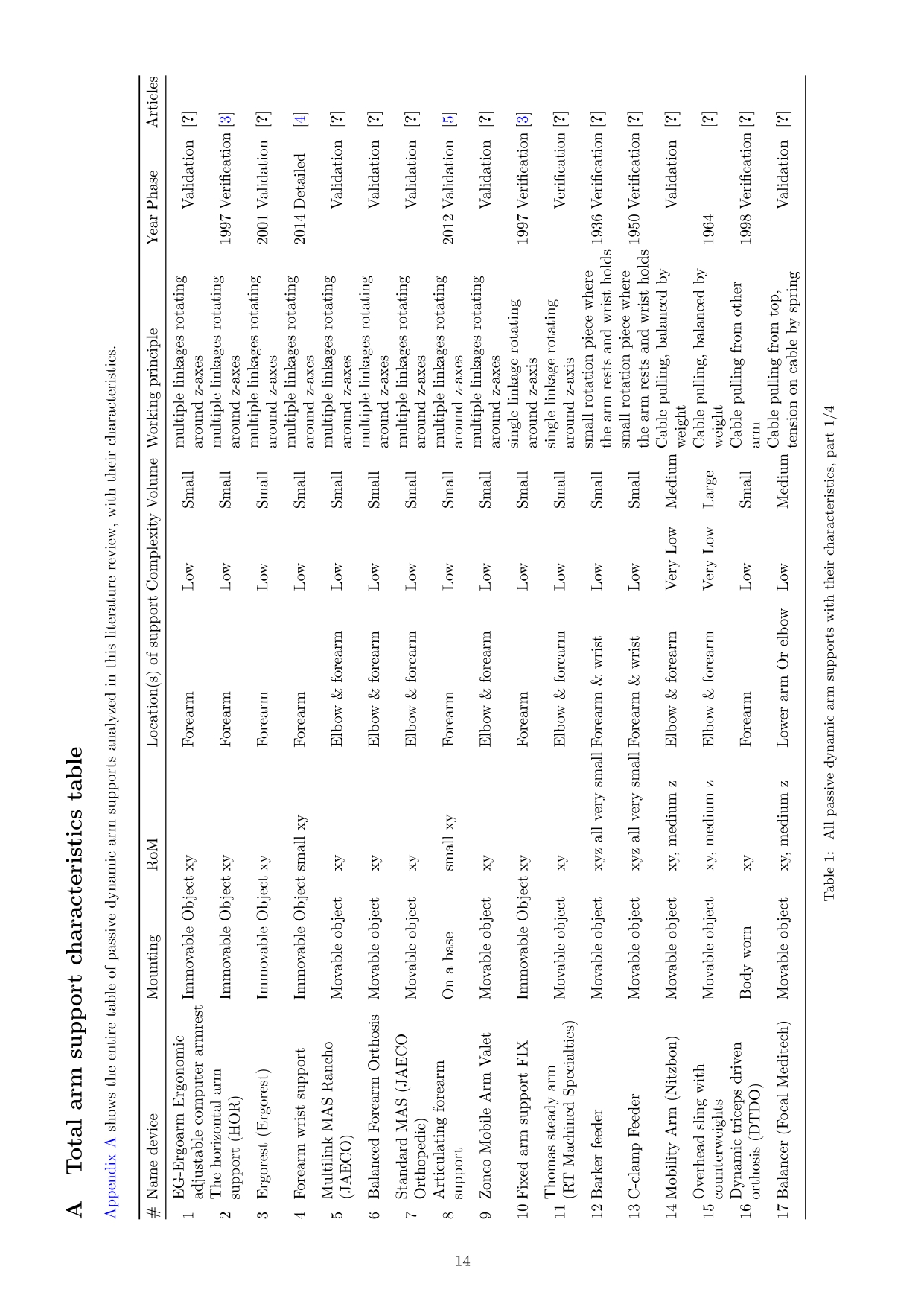


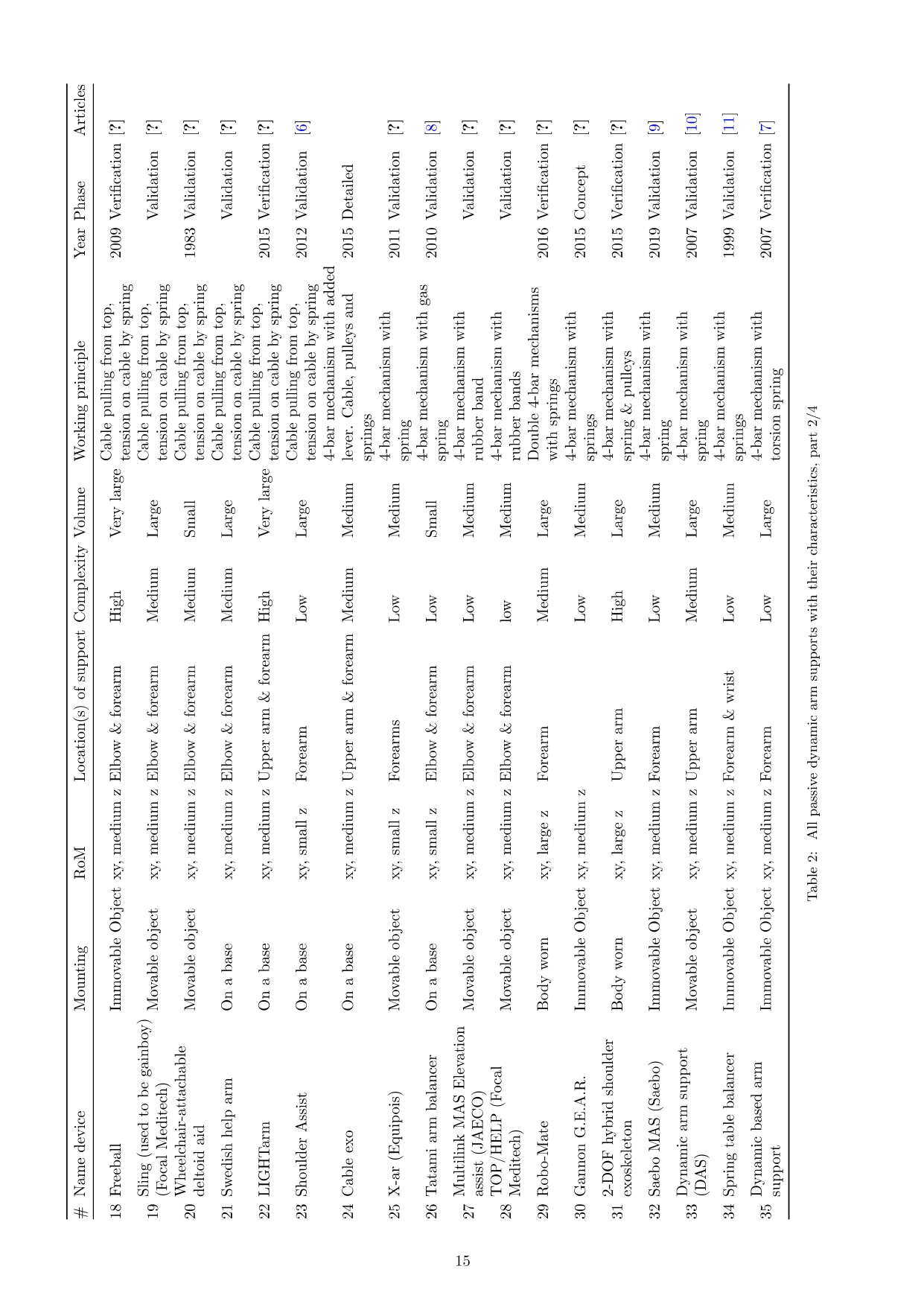


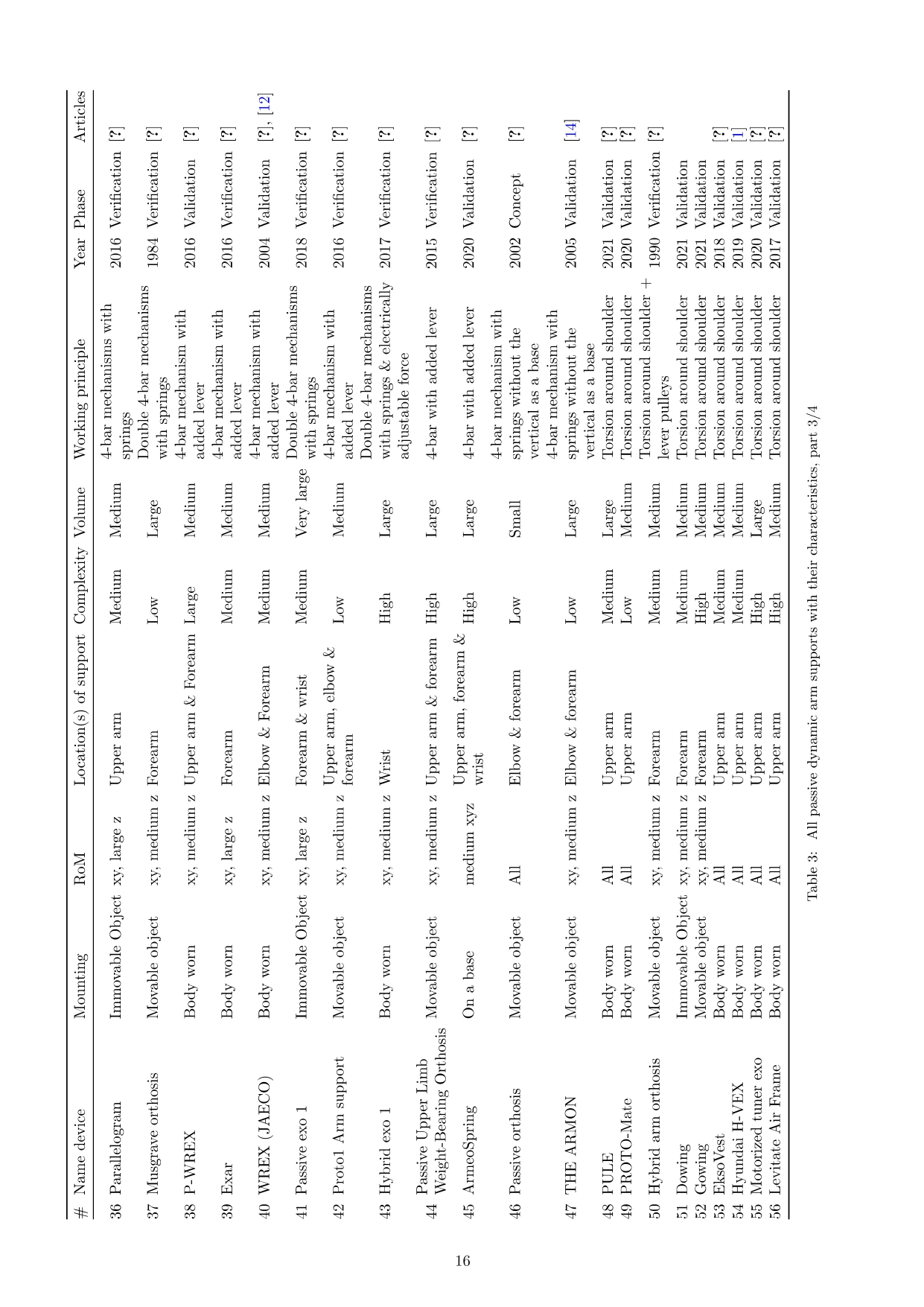


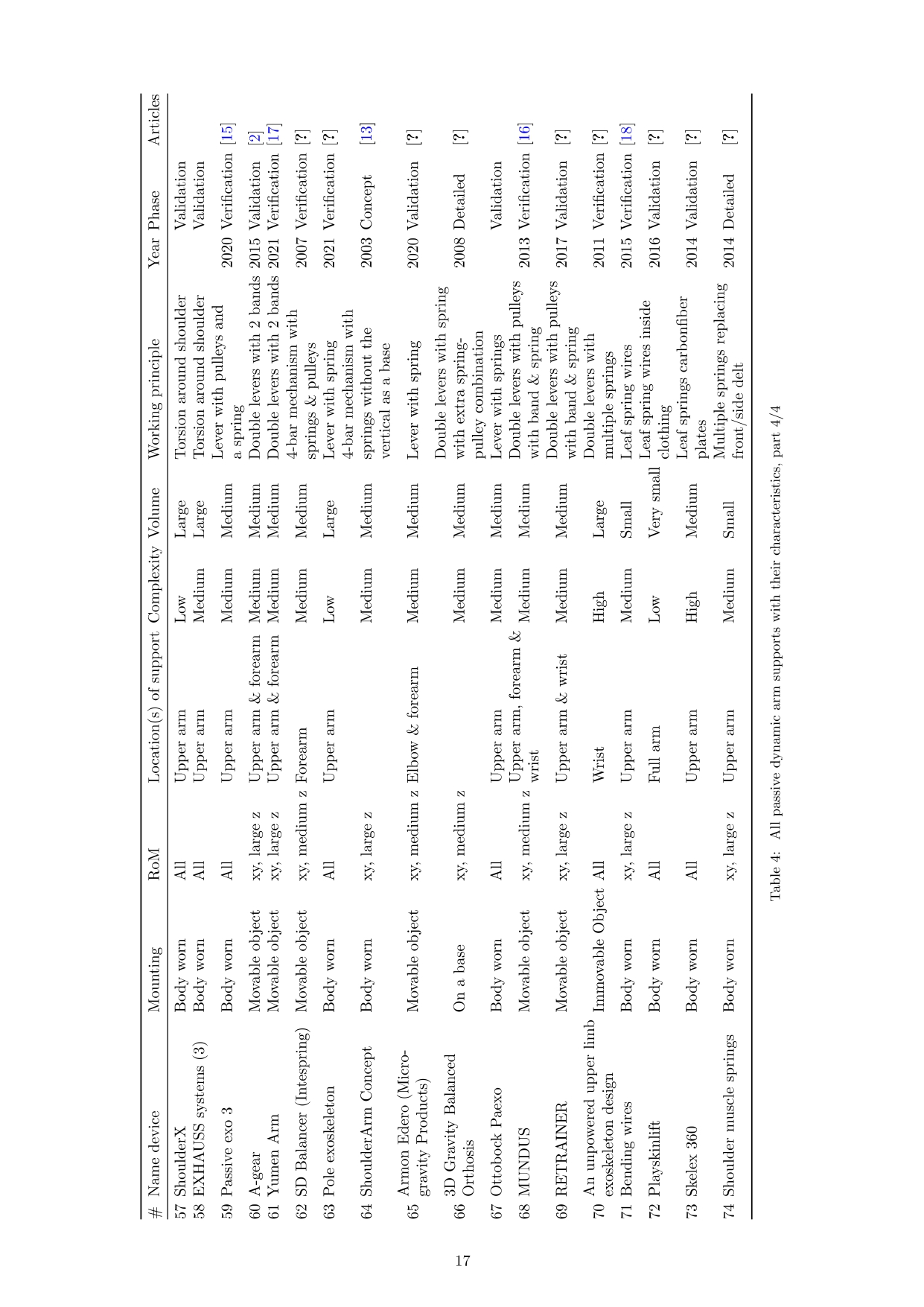

Supplement: Supplementary file 1 — Supplementary file1 (DOCX 4664 KB) [file 11701_2024_1820_MOESM1_ESM.docx]
