# Supplementary material for: Design and evaluation of a smart passive dynamic arm support for robotic-assisted laparoscopic surgery
Source: J Robot Surg. 2024 Feb 10;18(1):71. doi: 10.1007/s11701-024-01820-1 (PMC10858817; doi:10.1007/s11701-024-01820-1)
Supplement: Supplementary file 2 — Supplementary file2 (DOCX 1242 KB) [file 11701_2024_1820_MOESM2_ESM.docx]

**Supplemental File 2 the development of a simple contactless rotation sensor**

To track the opposition of the arms, new rotations sensors were developed based on the magnetic hall effect. In the final design of the Arm supports, there are three sensors per arm, able to measure all translations of the arm pad within the working area. They are placed on the rotational joints of the system, to measure the angle between links. The sensors function by moving a magnet across a hall sensor, which detects the magnetic field strength. This is implemented in two ways. Sensor A and B, as seen in Figure S2.1 and S2.2 and, work by attaching the magnet to one of the links and attaching the hall sensor to the other link. As the links rotate with respect to one another, the magnet moves along the hall sensor at a distance of 1 mm. Sensor C works differently, as it uses a lever that slides on a cam wheel. The magnet is attached to the lever and moves along the hall sensor in the same housing. The camwheel is attached to the other link. The reason for this different working principle is that sensor C must be able to measure a much larger range of angles compared to sensors A and B, which is not possible with the setup used in sensors A and B. All the sensor housings were 3D-printed, and the lever was made from a nylon cable tie. The sensors were powered and read by an Arduino Uno, which was connected to a laptop.


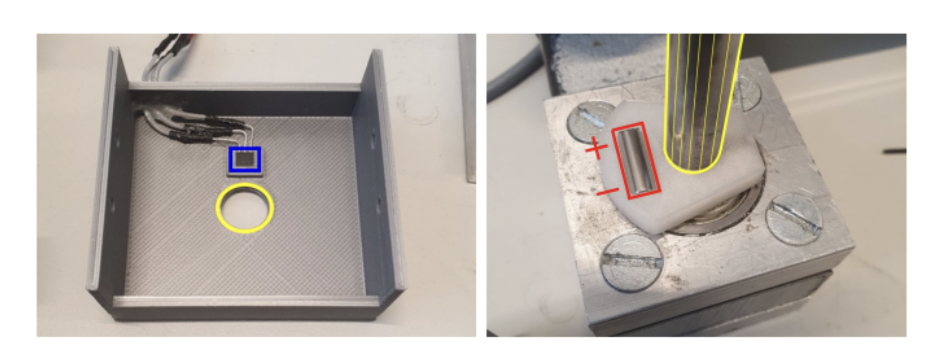


**Figure S2.1: Setup of sensor A. The hall sensor is indicated in blue, the magnet is indicated in red. The left part slides upside down with the yellow hole over the yellow shaft on the right, to ensure that the magnet moves along the hall sensor**


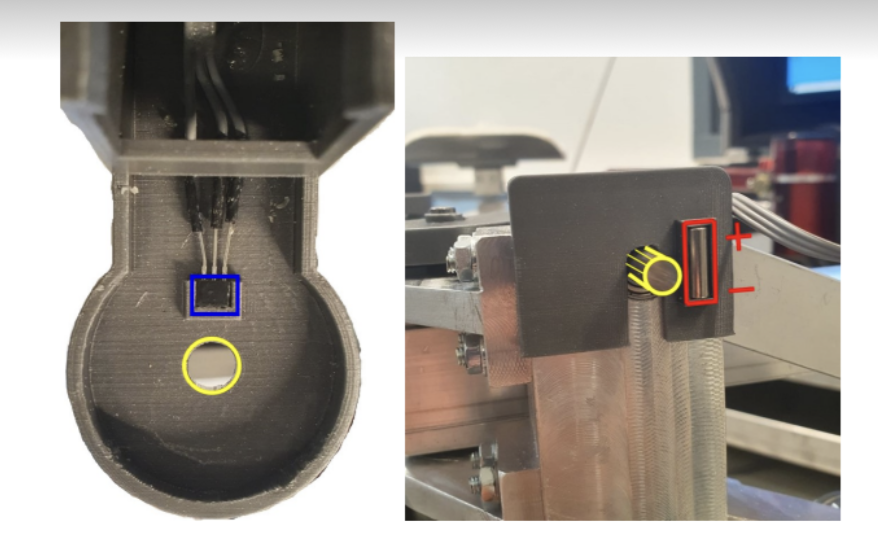


**Figure 2.3: Setup of sensor B. The hall sensor is indicated in blue, the magnet is indicated in red. The left part slides with the yellow hole over the yellow shaft on the right, to ensure that the magnet moves along the hall sensor**

To get accurate readings from the sensors on the position of the arm supports, the sensors had to be calibrated first (Figure S2.4). This was done by measuring the angles between the links throughout their ranges and noting the sensor value dis- played by Arduino at each angle. With more scientifically used programming software MATLAB, this data was fitted to polynomials to be able to determine angles when reading sensor values, as seen in Figure S2.4. These angles were used in combination with the dimensions of the system to calculate the position of the arm support pad in regard to the steel base on which the arm supports were mounted. This created six discrete sensor values of the sensors every 0.025 seconds. With a known relation between sensor output and rotation angle and the relation between rotation angles and linkages of the arm support, the position of the arm support in space can be calculated.


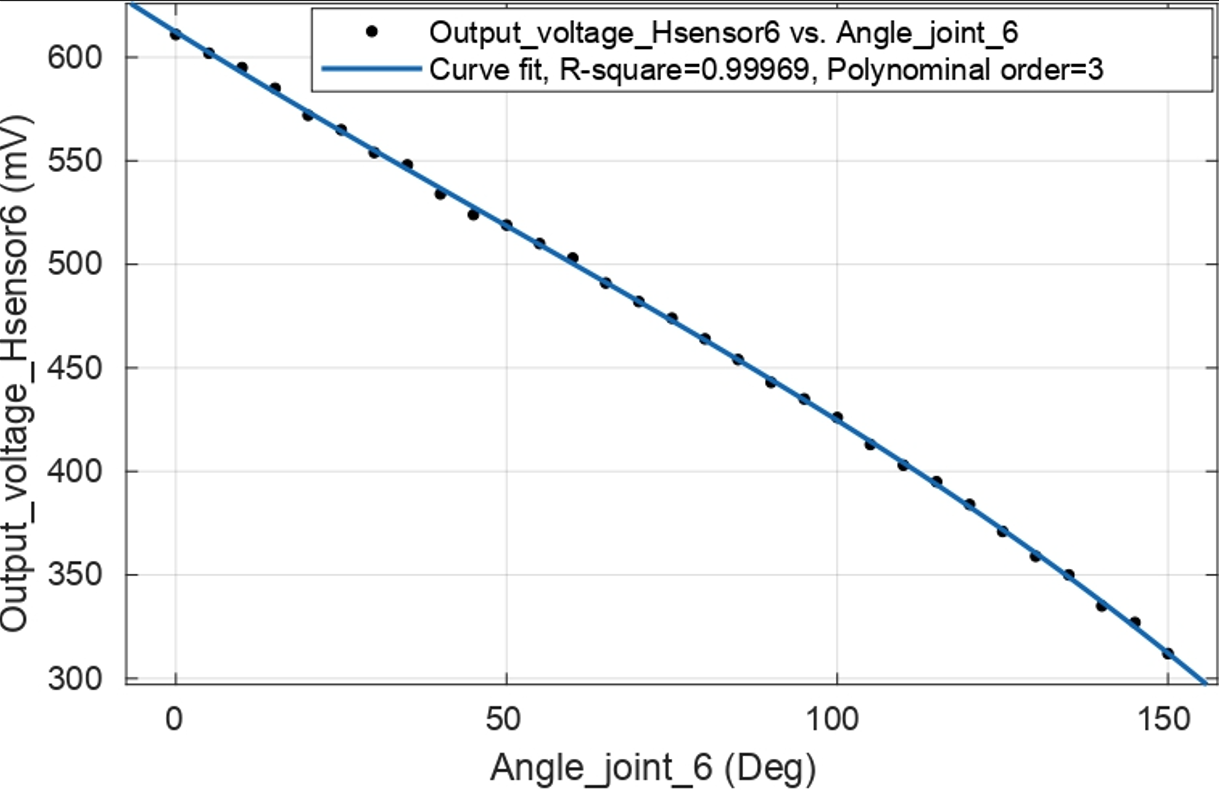


**Figure S2.3: Curves fit example of the output of one of the sensors that show a very nice fit along the relevant date range. The fitting formula for the 3th order polynomial is calculated as** **Angle joint =-0.0001OvH^3+0.0036OvH^2-1.9905OvH+612.2345**
