# Supplementary material for: Design and evaluation of a smart passive dynamic arm support for robotic-assisted laparoscopic surgery
Source: J Robot Surg. 2024 Feb 10;18(1):71. doi: 10.1007/s11701-024-01820-1 (PMC10858817; doi:10.1007/s11701-024-01820-1)

**Supplemental File 3 experimental validation**

Study “Experimental Validation of a dynamic arm support”

You are being invited to participate in a research study titled Validation study of a passive dynamic arm support for robotic laparoscopic surgery. This study is being done by Pim Schrijvershof from the TU Delft under the guidance of prof. dr. ir. T.Horeman.

The purpose of this research study is to validate the prototype that is built to measure changes in arm position. This help to determine the learning curve related to robotic surgery skills. This questionnaire will take you approximately 10 minutes to complete. The data will be used for a publication in an scientific journal. We will be asking you to perform a set task in the AdLab-RS robotic laparoscopy simulator for 8 times in a row while your arms are either supported or not supported by an arm support. During these tests, the position of your arms will be measured. Afterwards, we will ask you to fill in a question form on the perceived comfort of the entire activity. As with any online activity the risk of a breach is always possible. To the best of our ability your answers in this study will remain confidential. We will minimize any risks by converting the personal data into anonymous data, meaning that all names will be attached to a participant number, which can’t be traced back to the participants. The data will be stored on the project storage of the TU Delft. No sensitive data will be gathered from participants.

Your participation in this study is entirely voluntary and you can withdraw at any time. You are free to omit any questions. Within 2 weeks of the experiments your data can still be removed if wished for.

Thank you for taking part!

Pim Schrijvershof and Tim horeman

Email: p.schrijvershof@tudelft.nl
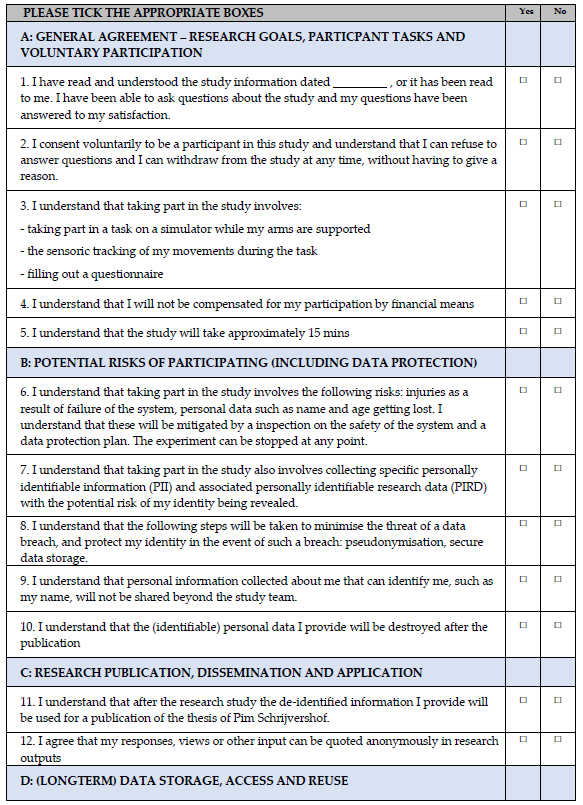


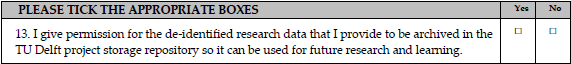


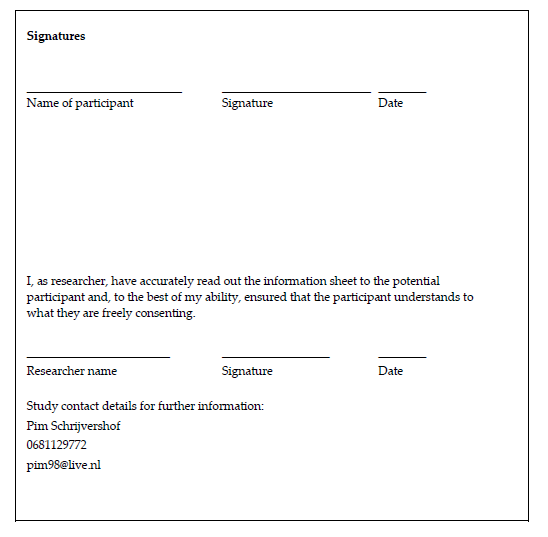


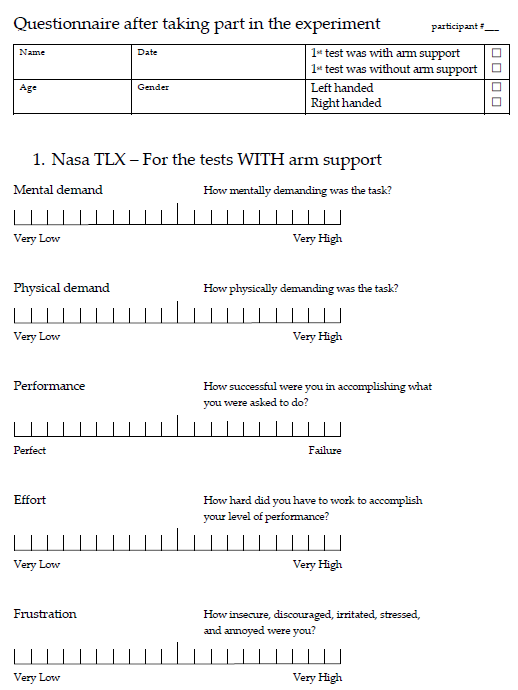


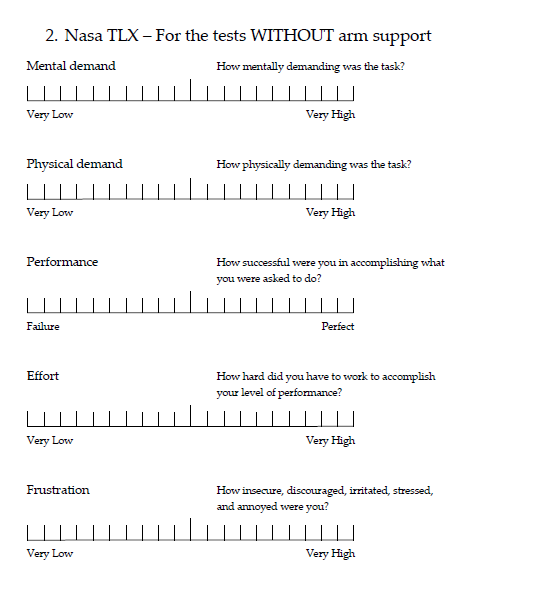


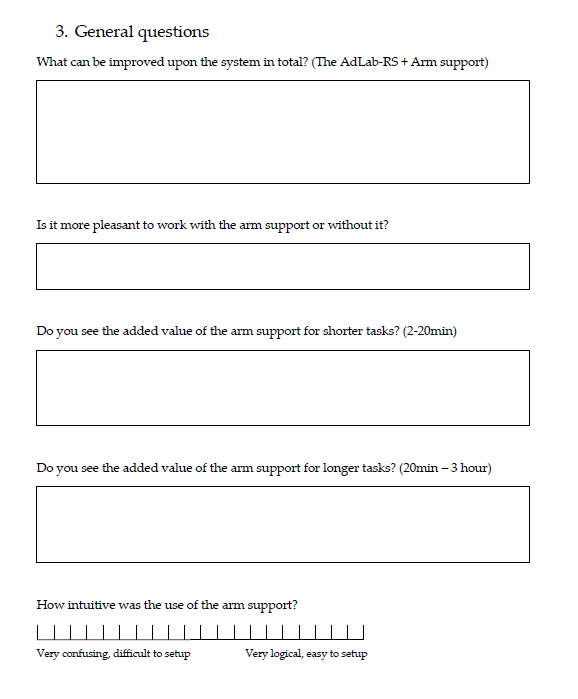

Supplement: Supplementary file 3 — Supplementary file3 (DOCX 294 KB) [file 11701_2024_1820_MOESM3_ESM.docx]
